# Microbial Community Shifts and Plant Performance Improvements Driven by *Bacillus firmus* in Pampa Agroecosystems

**DOI:** 10.1111/1758-2229.70315

**Published:** 2026-05-15

**Authors:** David Fagundes, Laís Mara Santana Costa, Alexandro Cagliari, Diego Prado de Vargas, Alexandre Rieger

**Affiliations:** ^1^ Postgraduate Program in Environmental Technology (PPGTA) University of Santa Cruz do Sul (UNISC) Santa Cruz do Sul Rio Grande do Sul Brazil; ^2^ ConnectBIO, Soluções Biotecnológicas Santa Cruz do Sul Rio Grande do Sul Brazil; ^3^ State University of Rio Grande do Sul (UERGS) Santa Cruz do Sul Rio Grande do Sul Brazil

**Keywords:** *Bacillus firmus*, biological nematode control, microbial community dynamics, Pampa biome, rhizosphere microbiota, soil microbial ecology, soybean

## Abstract

The impact of *Bacillus firmus* –based bionematicides on rhizosphere microbiota and plant performance remains poorly understood in complex agroecosystems. This study evaluated the effects of *B. firmus* application on soil microbial communities, nematode dynamics, and soybean productivity in the Pampa biome. Our results demonstrate that *B. firmus* significantly modulates the composition and diversity of soil microbiota, with effects varying across locations and over time. Treated areas exhibited shifts in bacterial communities, including increased abundance of beneficial taxa, while fungal diversity tended to decrease, likely due to the combined effect of the fungicide used in seed treatment. Despite persistent populations of target nematodes such as *Pratylenchus brachyurus* and *Heterodera glycines* , treated areas showed reduced root infestation in specific stages and locations. Importantly, the application of *B. firmus* consistently enhanced soybean shoot and root growth, resulting in productivity gains of 6%–7% across all sites. These findings reveal that *B. firmus* not only contributes to plant growth promotion but also induces significant, yet context‐dependent, shifts in rhizosphere microbial communities. The study highlights the ecological complexity of microbial responses to biocontrol agents and underscores the importance of integrating microbiome dynamics into sustainable nematode management strategies in agroecosystems.

## Graphical abstract

The study shows that *Bacillus firmus* ‐based bionematicides modulate soil microbiota and enhance soybean growth in the Pampa biome. Effects vary by location and time, with increased beneficial bacteria, reduced fungi, and decreased root nematode infestation. Overall, *B. firmus* improved productivity by 6%–7%, supporting sustainable nematode management strategies.

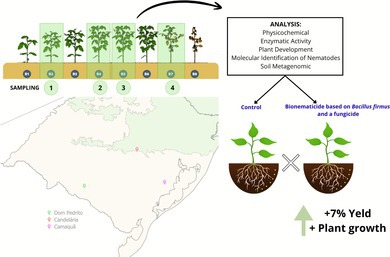

## 1 Introduction

Phytoparasitic nematodes represent a major constraint to global agricultural productivity, with estimated annual crop losses exceeding US$358 billion worldwide (Zhou et al. 2024). These organisms impair root system integrity, disrupt water and nutrient uptake, and cause yield reductions across a wide range of economically important crops (Beeman and Tylka 2018; Jones et al. 2013). Although more than 4100 nematode species have been described, a relatively small fraction is responsible for severe agricultural damage, notably *Heterodera glycines* , *Meloidogyne* spp., and *Pratylenchus* spp., which affect soybean, maize, and cotton production systems worldwide (Elhady et al. 2017; Engelbrecht et al. 2018; Jones et al. 2013). In Brazil, particularly in soybean‐growing regions, nematode infestations result in substantial economic losses, with yield reductions commonly ranging from 10% to 30% and, in heavily infested areas, reaching up to 85% reductions in key yield components (CONAB 2022; Lima et al. 2017). The nonspecific nature of aboveground symptoms often delays diagnosis and hampers timely management interventions (Jones et al. 2013; Wendimu 2022).

Historically, nematode control has relied predominantly on synthetic chemical nematicides, including carbamates, organophosphates, and soil fumigants such as methyl bromide (Beeman and Tylka 2018; Elhady et al. 2017; Susic et al. 2020). Despite their efficacy, these compounds are associated with serious environmental and toxicological concerns, including groundwater contamination, high toxicity to non‐target organisms, and the emergence of resistant nematode populations under repeated exposure (Engelbrecht et al. 2018; Ristaino and Thomas 1997). Consequently, increasing regulatory restrictions—such as the global ban on methyl bromide under the Montreal Protocol and the limitations imposed by European Regulation EC 1107/2009—have drastically reduced the availability of effective chemical nematicides (Oka 2010; UNEP 2020; EPRS 2018). These constraints, coupled with growing societal demand for safer and more sustainable food production systems, have intensified the search for alternative nematode management strategies.

Within this context, bionematicides have emerged as promising tools for integrated nematode management, offering reduced environmental impact and greater compatibility with sustainable agricultural practices (Ghahremani et al. 2019; Sahu et al. 2018; Shrestha 2022). These products are based on living microorganisms or their bioactive metabolites and may suppress nematodes through multiple mechanisms, including direct parasitism, antibiosis, competition, and induction of plant defense responses (Kohl et al. 2019). Among these biological agents, species of the genus *Bacillus*—notably *B. firmus* , *B. thuringiensis* , and *B. subtilis* —have attracted particular attention due to their ability to produce a diverse array of nematicidal compounds, such as cyclic lipopeptides (surfactins, iturins, fengycins), hydrolytic enzymes, and volatile organic compounds (Li et al. 2015; Engelbrecht et al. 2018; Mendis et al. 2018; Susic et al. 2020). The multiplicity of these modes of action not only enhances control efficacy but also reduces the likelihood of resistance development (Ghahremani et al. 2020; Gu et al. 2022; Kohl et al. 2019).

Beyond direct nematode suppression, *Bacillus*‐based bionematicides may contribute to plant health by inducing systemic resistance mediated by jasmonic acid‐ and ethylene‐dependent signalling pathways, leading to the upregulation of defence‐related genes and improved tolerance to biotic stress (Ghahremani et al. 2020; Schrimsher 2013; Beeman and Tylka 2018). Additionally, successful rhizosphere colonisation by these bacteria can improve soil biological functioning and nutrient availability, even in the absence of pathogen pressure (Backer et al. 2018). However, the field performance of bionematicides remains variable and is strongly influenced by abiotic conditions such as soil pH, moisture, temperature, texture, and organic matter content, as well as biotic interactions with native soil microbiota (Beeman et al. 2019; Kohl et al. 2019; Zhang et al. 2017). For example, the establishment of *B. firmus* populations has been shown to be limited in acidic soils or under water stress, reducing its protective efficacy (Beeman et al. 2019; Gu et al. 2022).

An important yet insufficiently explored factor affecting bionematicide performance is their interaction with other agrochemicals routinely applied in crop production systems. Seed‐applied fungicides may exert antagonistic or synergistic effects on biocontrol agents, depending on their chemical composition and mode of action (Beeman et al. 2019; Villavicencio‐Vásquez et al. 2025). These interactions are particularly relevant in integrated pest and disease management programs, where multiple inputs are applied concurrently or sequentially, potentially influencing rhizosphere microbial dynamics and biological control outcomes.

The Pampa biome of southern Brazil provides a relevant ecological and agronomic setting for investigating such interactions. Characterised by sandy soils, high erosion susceptibility, and a temperate climate with marked seasonality, this biome hosts distinct microbial communities but has undergone rapid agricultural intensification in recent decades (Overbeck et al. 2007; Barbosa et al. 2023; Overbeck et al. 2024). Evidence suggests that intensive management practices have altered nematode community structures, favouring generalist phytoparasitic species while reducing populations of free‐living nematodes involved in nutrient cycling and natural biological regulation (Machado et al. 2022; Pothula et al. 2022).

Against this backdrop, the objective of this study was to evaluate the effects of a *Bacillus firmus* ‐based bionematicide (strain I‐1582), applied in combination with a fungicide formulation containing fipronil, pyraclostrobin, and thiophanate‐methyl, on phytoparasitic nematode control and soil health in soybean cultivation areas within the Pampa biome. We hypothesized that interactions between these compounds could result in synergistic or antagonistic effects mediated by their compatibility with rhizosphere microbiota (Engelbrecht et al. 2018). To address this hypothesis, we assessed agronomic performance, soil biochemical indicators, nematode population dynamics, and qualitative and quantitative changes in rhizosphere microbial communities using high‐throughput metagenomic sequencing. By integrating agronomic, biochemical, and microbiological approaches, this study aims to advance understanding of bionematicide performance under realistic field conditions and to support the development of more resilient and sustainable nematode management strategies in soybean‐based agroecosystems.

## 2 Materials and Methods

### 2.1 Selection of Experimental Areas

**FIGURE 1:**
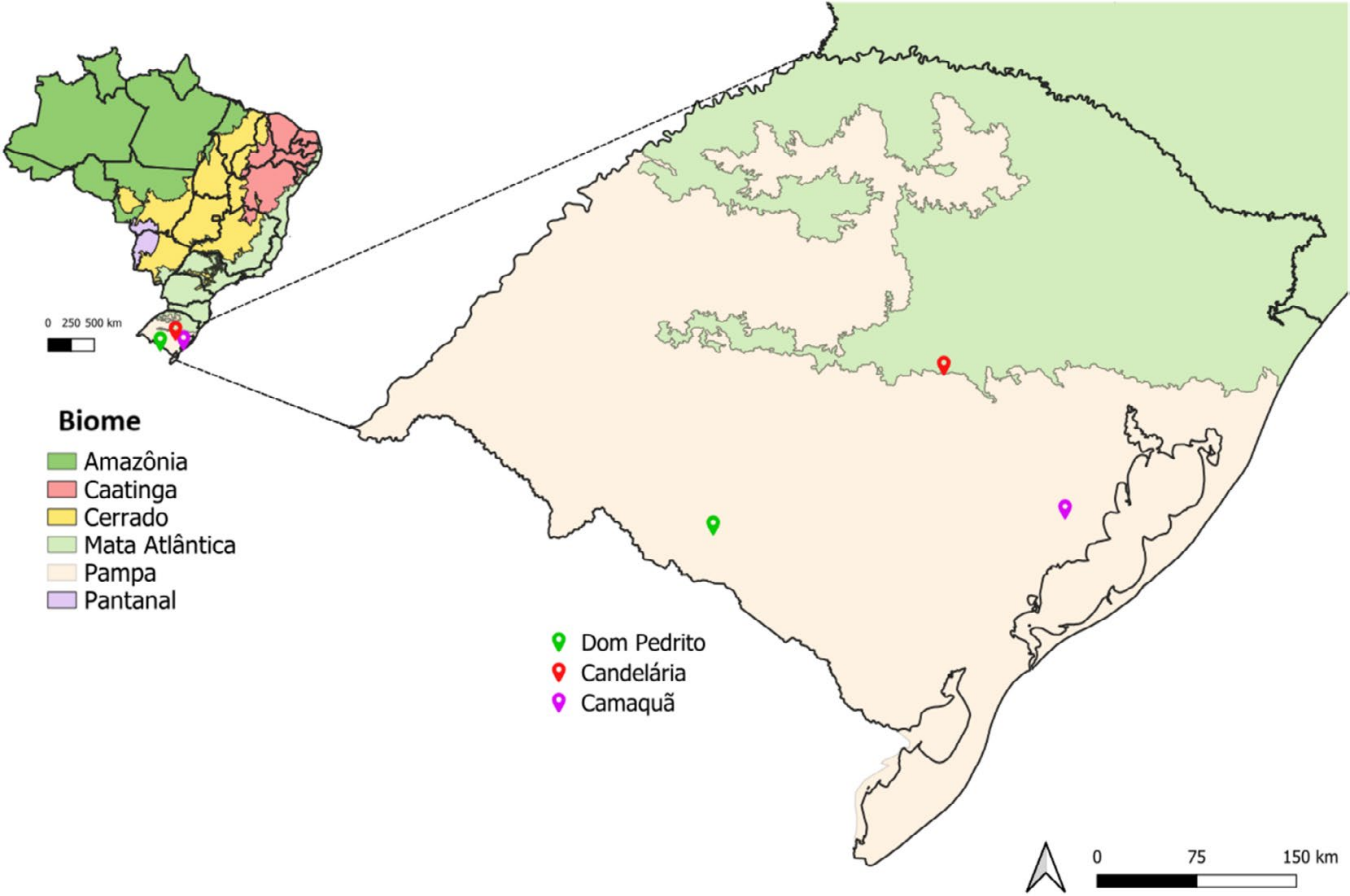
Location of the municipalities of Dom Pedrito, Candelária, and Camaquã in the state of Rio Grande do Sul, Brazil, where the study was conducted. Coloured markers indicate the sampled municipalities, situated within the Pampa biome (shown in beige). The larger map details the state of Rio Grande do Sul, while the smaller inset map shows the distribution of Brazilian biomes.

The study was conducted in three municipalities within Rio Grande do Sul, Brazil: Dom Pedrito, Candelária, and Camaquã, representing an edaphoclimatic gradient characteristic of the Pampa biome. The geographic location of the municipalities sampled in this study, within the state of Rio Grande do Sul, Brazil, is shown in Figure 1. The Pampa biome, which extends across southern Brazil and into Uruguay and Argentina, constitutes one of the most biodiverse grassland ecosystems globally, harbouring more than 3000 plant species along with rich associated fauna (Barbosa et al. 2023; Michel and Overbeck 2024; Pillar et al. 2009).

Municipality selection was based on the representativeness of distinct geomorphological units and predominant soil classes within the Pampa of Rio Grande do Sul. Dom Pedrito, located in the Campanha region, is characterised by Ebanic Vertisol soils, while Candelária, situated in the Central Depression, predominantly features Argisols. Camaquã, positioned within the Coastal Plain, is distinguished by the occurrence of Umbric Red‐Yellow Dystrophic Argisol soils (Streck et al. 2018).

### 2.2 Treatments

The study compared untreated soybean seeds (Control) with seeds subjected to a pre‐planting chemical–biological treatment involving a fungicide–insecticide formulation followed by a bionematicide. During seed treatment, the fungicide–insecticide mixture containing fipronil, pyraclostrobin, and thiophanate‐methyl was first applied at a rate of 2 mL kg^−1^, ensuring uniform coating. Subsequently, a biological nematicide based on *Bacillus firmus* was applied at 1 mL kg^−1^, completing the sequential treatment.

This chemical formulation is commonly used in soybean seed treatments to protect against early‐season diseases and insect pests. While its effectiveness in protecting soybean seedlings against various pathogens and insects is well documented, its impact on plant physiology and potential phytotoxicity, particularly when combined with other seed treatments, warrants further investigation. Several studies have reported that such formulations can affect nitrogen fixation activity in soybean plants, emphasizing the need for balanced approaches that maximise crop yield while minimising environmental risks (Sartori et al. 2023; Zuffo et al. 2021).

The combined application of the *Bacillus firmus* ‐based bionematicide and the chemical seed treatment presents a complex scenario for investigation. Although both aim to enhance crop protection, their interaction may result in either synergistic effects or increased phytotoxicity. Research specifically addressing the interactions between biological and chemical seed treatments remains limited, representing a critical gap that must be addressed to develop more effective and sustainable soybean production practices.

### 2.3 Sampling

**FIGURE 2:**
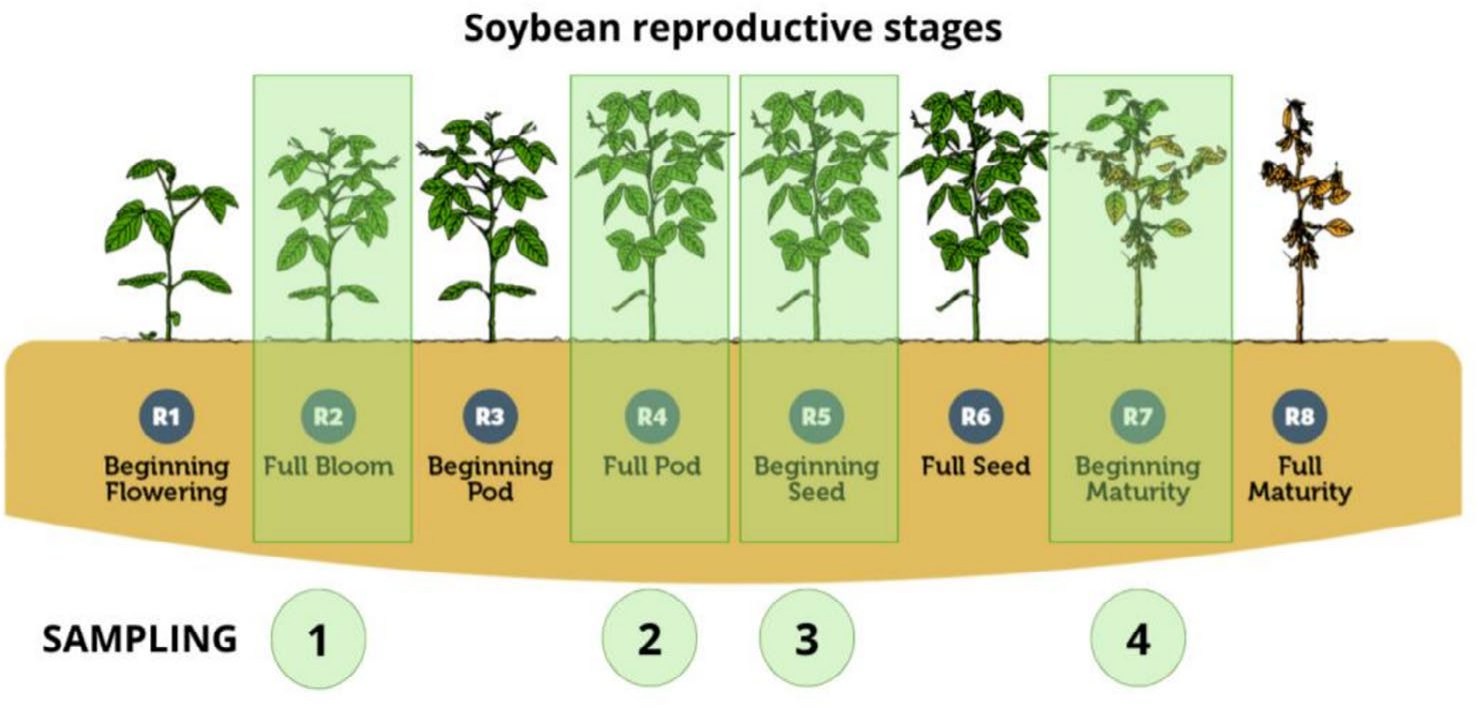
Reproductive stages of soybean and sampling points of the study. Soil and root samples were collected at the phenological stages R2 (full flowering), R4 (pod formation), R5 (beginning of seed filling), and R7 (beginning of maturation), highlighted in green.

To obtain comprehensive data on treatment impacts, soil and root samples were collected at four distinct phenological stages of the soybean growth cycle: R2 (full flowering), R4 (pod formation), R5 (beginning of seed filling), and R7 (beginning of maturation), as illustrated in Figure 2.

The classification of productive environments was derived from historical NDVI time series obtained from Sentinel‐2 MSI imagery (10 m resolution) covering the 3–5 years preceding the experiment. Image processing was conducted on the Google Earth Engine (GEE) platform using Sentinel‐2 Level‐2A products, which include atmospheric correction via Sen2Cor. NDVI was calculated from the near‐infrared and red spectral bands. To delineate productive environments, we employed a hybrid approach consisting of an initial k‐means segmentation to reduce within‐class variability, followed by an unsupervised clustering procedure (*k* = 3) to generate three strata representing High, Medium, and Low productivity zones. Visual and agronomic validation of the resulting clusters was performed using historical yield maps provided by the producer (Lasaponara et al. 2022).

Soil and root sampling was performed at all georeferenced points within each plot following standardised procedures. Five subsamples were collected per area, each obtained from a 20 to 30 cm depth using a V‐shaped soil cut, maintaining natural soil moisture and preventing root desiccation. The five subsamples were homogenised to form a single composite sample per area, consisting of approximately 1 kg of soil and, when present, 50–100 g of roots. Samples were immediately sealed in labelled plastic bags, kept in a cool and shaded environment, and transported promptly to the laboratory. When short‐term storage was necessary, samples were maintained at 4°C to preserve microbial integrity prior to processing (Filizola et al. 2006). All sampling procedures were conducted by a technical team to ensure consistency and precision. Collected samples were carefully packaged and sent to specialised laboratories for subsequent analyses.

### 2.4 Physicochemical Analysis

Comprehensive physicochemical soil analysis was conducted to understand treatment effects on soil composition and structure. Chemical analysis quantified essential macro and micronutrients, including nitrogen, phosphorus, potassium, calcium, magnesium, and trace elements like zinc and iron, which directly influence crop productivity. Physical evaluation determined soil texture by measuring the relative proportions of silt, sand, and clay particles present in samples.

The concentrations of phosphorus, copper, zinc, sodium, boron, potassium, and iron were quantified using the Mehlich‐1 extraction method (Mehlich 1978). Soil organic matter was determined via sulfodichromate oxidation (Walkley and Black 1934). Clay content was obtained through a densimetric sedimentation approach (Gee and Bauder 1986). Exchangeable calcium, magnesium, aluminium, and manganese were extracted using NH_4_Cl (Hendershot et al. 1993), while available sulfur was assessed using the ammonium acetate extraction method (Bao 2000). Soil pH in water and SMP buffer index were determined potentiometrically (potentiometer model HI5521, Hanna Instruments) (Thomas 1996). The sand, silt, and clay fractions were characterised using the Bouyoucos hydrometer method (Bouyoucos 1962).

The analyses were conducted in a certified laboratory participating in the Official Network of Soil and Plant Tissue Analysis Laboratories of Rio Grande do Sul and Santa Catarina (ROLAS), which ensures analytical standardisation and continuous performance monitoring across accredited facilities (SBCS 2025). The laboratory is also enrolled in the Embrapa Soils Quality Analysis Program for Fertility Laboratories (PAQLF), a national proficiency testing scheme that evaluates analytical accuracy and awards quality certification based on interlaboratory performance (Embrapa Solos 2025).

Given the reduced sample size available for each treatment, considering that one analysis was performed for each soil sample received from the field, the assumptions of normality and homogeneity of variances could not be reliably established. To ensure statistical rigour and avoid bias arising from asymmetric distributions or potential outliers, the non‐parametric Mann–Whitney U test (Mann and Whitney 1947) was applied to compare the treated and control groups. This rank‐based test is suitable for small samples and does not rely on parametric assumptions, providing a more robust and consistent assessment of differences between treatments.

### 2.5 Enzymatic Activity Analysis

Soil biological activity, a critical indicator of soil health, was assessed through enzyme analyses measuring the activity levels of specific enzymes involved in nutrient cycling. The enzymes analysed were β‐glucosidase (carbon cycle), arylsulfatase (sulfur cycle), *N*‐acetylglucosaminidase (NAGase; nitrogen cycle), and acid phosphatase (phosphorus cycle).

Enzymatic activities were measured following the ConnectBIO enzymatic analysis protocol, adapted from Tabatabai (1994). Briefly, 5 g of soil were homogenised in 10 mL of 50 mM acetate buffer (pH 5.0–5.5). Aliquots of 150 μL were dispensed into deepwell plates in duplicate and incubated with 5 mM enzymatic substrates (Sigma–Aldrich): *p* nitrophenyl β‐D‐glucopyranoside for β‐glucosidase, potassium 4‐nitrophenyl sulfate for arylsulfatase, 4‐nitrophenyl N‐acetyl‐β‐D‐glucosaminide for NAGase, and 4‐nitrophenyl phosphate disodium salt hexahydrate for acid phosphatase. Reactions were carried out at 22°C–24°C with continuous agitation for 2 h (β‐glucosidase and NAGase), 4 h (arylsulfatase), and 1 h (acid phosphatase). After incubation, plates were centrifuged at 5000 g for 5 min, and 100 μL of the supernatant were transferred to microplates containing 1 M NaOH to stop the reactions. Absorbance was measured at 410 nm using a microplate reader (Synergy HTX Multi‐Mode Reader, BioTek Instruments, USA) and activities were normalised to soil dry mass based on a nitrophenol calibration curve.

Enzymatic activity levels in treated and untreated areas were compared to evaluate the treatment's influence on soil biological processes and nutrient availability. Activity dynamics over time were assessed considering different sampling periods and production environments. One‐way ANOVA was used to investigate variations in enzymatic activity based on temporal factors and environmental characteristics.

### 2.6 Plant Development Analysis

For plant development assessment, 10 plants per treatment were randomly selected along a systematic walk in a zigzag trajectory across the central region of each plot, avoiding border rows to minimise edge effects and ensure data independence. Analyses were conducted at 15 and 30 days after emergence to characterise early growth dynamics. Morphological and biometric parameters measured included plant population density, aerial part and root system lengths, and fresh biomass of vegetative structures.

Plant stand determination was performed by counting and analysing population density within experimental areas to verify seedling establishment uniformity and identify any initial development failures. Aerial part length was measured using a ruler, considering the distance from the base to the plant apex. Root length was obtained after careful soil extraction, cleaning, and measurement of the primary root and longest branches. Fresh biomass of aerial parts and roots was determined immediately after collection using a precision balance.

Due to technical constraints, fresh biomass measurements 15 days after emergence in Dom Pedrito were not conducted. For statistical analysis, parameter values were compared between Control and Treatment in each location using the Mann–Whitney test to detect statistically significant differences.

### 2.7 Molecular Identification of Plant‐Parasitic Nematodes

Real‐time PCR (qPCR) was employed for diagnosing plant nematode presence in the analysed areas. This molecular technique allows precise detection and quantification of specific DNA sequences, with the extraction and amplification method ensuring accurate identification regardless of environmental conditions or nematode developmental stage.

To assess nematode presence and abundance, both soil and root samples from the same areas were analysed to verify whether soil nematodes had indeed infected soybean roots. Target nematode species in the research were: *Rotylenchulus reniformis*, *Heterodera glycines* , *Helicotylenchus dihystera*, *Pratylenchus brachyurus* , and *Meloidogyne* spp.

Quantitative PCR (qPCR) assays were performed using the PowerUp SYBR Green Master Mix (Thermo Fisher Scientific), in accordance with the manufacturer's recommendations. Reactions were prepared in optical plates with a final volume of 10 μL, containing the SYBR Green master mix, gene‐specific forward and reverse primers at optimised concentrations, and template DNA diluted in nuclease‐free water. Amplifications were carried out on a QuantStudio 5 Real‐Time PCR System (Applied Biosystems). The thermal cycling program was run in fast mode and consisted of an initial UDG activation step at 50°C for 2 min, followed by polymerase activation and initial denaturation at 95°C for 2 min. Amplification was then performed for 45 cycles, each comprising a denaturation step at 95°C for 1 s and a combined annealing/extension step at 60°C for 30 s, during which fluorescence data were collected. Melting curve analysis was performed at the end of each run to verify amplicon specificity. All samples were analysed in technical duplicate, and appropriate positive and negative controls were included in each run.

### 2.8 Soil Metagenomic Analysis

Metagenomic analysis of soil microbial communities was conducted using next‐generation sequencing (NGS) technology on the Illumina platform. Analysis focused on identifying taxonomic groups common across all samples or exclusive to specific locations, facilitating comparisons between treated and untreated zones. Only genera with more than 1% relative abundance were considered statistically significant for analysis, a threshold chosen to minimize the influence of low‐abundance taxa prone to higher error rates and variability (Cena et al. 2021; McMullen et al. 2020).

The extraction of soil DNA was performed using the MagMAX Microbiome Ultra Nucleic Acid Isolation Kit (Thermo Fisher Scientific), following the manufacturer's instructions.

High‐throughput sequencing and bioinformatic analyses were performed by Biome4All (São Paulo, Brazil). Bacterial communities were characterised by targeting the 16S rRNA gene (V3–V4 region), while fungal communities were assessed using the ITS2 region (Gardes and Bruns 1993; Woo et al. 2008). Amplicons were sequenced on an Illumina platform, generating paired‐end reads with an average length of 250 bp.

Raw reads were processed using the QIIME2 pipeline (Bolyen et al. 2019). Adapter and primer sequences were removed, and fungal ITS data were further processed using the ITSxpress2 plugin for region extraction and quality control. Denoising, paired‐end merging, and chimera removal were performed with the DADA2 algorithm implemented in QIIME2, using default parameters, resulting in amplicon sequence variants (ASVs) (Callahan et al. 2016). Non‐target sequences, including chloroplast and mitochondrial reads, were removed prior to downstream analyses.

Taxonomic classification was conducted using a naïve Bayes classifier, with bacterial ASVs assigned against the Greengenes database and fungal ASVs against the UNITE database, following standard confidence thresholds. Downstream interpretation and visualisation of microbial community profiles were performed using the Biome4all Agri‐Analysis platform (v.2.1). Raw read counts were normalised by total‐sum scaling, converting counts to relative abundances prior to downstream analyses. No rarefaction was applied.

To investigate the effect of the treatment on the relative abundance of the dominant microbial genera, relative abundance data were first adjusted by adding a small pseudocount (0.001) to account for zero values. Data were then transformed using the centered log‐ratio (CLR) transformation to address the compositional nature of microbiome data and allow statistical analysis in Euclidean space (Gloor et al. 2017). Following transformation, treated and control samples were compared using the Wilcoxon signed‐rank test, considering paired observations within each sampling event. For these analyses, data from different cities were pooled in order to increase the effective sample size for statistical testing, while preserving the pairing between treated and control samples. Given the limited number of observations, the analyses were interpreted as exploratory, focusing on the direction and magnitude of treatment‐associated changes in the relative abundance of the dominant genera.

Diversity indices were calculated using the Python programming language with Pandas, NumPy, Scipy, Scikit‐bio, and Matplotlib libraries for data organisation, diversity calculation, and result visualisation. Alpha diversity was assessed using the Shannon index to quantify microbial community diversity. Differences in Shannon index values between control and treated samples were evaluated using a paired Wilcoxon signed‐rank test (Wilcoxon 1945). To ensure sufficient sample size and statistical power, data from all locations were pooled, and comparisons were performed between treatment conditions within each sampling time independently.

For beta diversity analysis, community dissimilarities were primarily quantified using the Bray–Curtis index, which incorporates relative abundance information and is therefore more sensitive to shifts in dominant and subdominant taxa (Bray and Curtis 1957). Bray–Curtis dissimilarity matrices were used as the basis for all ordination and hypothesis‐testing procedures. Principal Coordinates Analysis (PCoA) was performed separately for each city to avoid conflation of independent dissimilarity spaces, allowing a robust visualisation of treatment‐ and sampling time–related patterns within each location, following standard distance‐based ordination approaches in community ecology. Differences in bacterial community composition were formally tested using permutational multivariate analysis of variance (PERMANOVA) based on Bray–Curtis distances (Anderson 2001), applying a sequential (Type I) model in which treatment and sampling time were evaluated in the order specified in the model, thereby partitioning variance cumulatively within each city (Legendre and Legendre 2012).

The Jaccard index, which is based solely on presence–absence data, was calculated as a complementary metric to qualitatively assess changes in community membership (Magurran 2004). Jaccard dissimilarities were used exclusively for descriptive comparisons, supporting the interpretation of whether observed differences were driven by shifts in relative abundances or by the gain or loss of taxa but were not included in the main inferential statistical analyses.

## 3 Results

### 3.1 Directional Yield Gains Under *Bacillus firmus* –Based Treatment Across Contrasting Environments

Analysis demonstrated consistent increases in soybean productivity across all evaluated locations following treatment with the *Bacillus firmus* ‐based bionematicide combined with the fungicide‐insecticide formulation (see Tables 1 and S1).

**TABLE 1:** Soybean yield (expressed in bags per hectare) in untreated (control) and treated plots across three locations in Rio Grande do Sul, Brazil.

Location	Treatment	Yield (bags/ha)
Dom Pedrito	Control	50.3
	Treated	53.2
	Percent increase	6%
Candelária	Control	52.6
	Treated	55.6
	Percent increase	6%
Camaquã	Control	89.6
	Treated	95.5
	Percent increase	7%

*Note:* 1 bag = 60 kg. Percent increase refers to the relative gain in yield observed in treated plots compared to the respective control within each location.

When yield was compared between control and treated plots (see Table 2), the two‐tailed Mann–Whitney *U* test did not detect statistical significance (*p* = 0.369). However, considering the a priori biological expectation of increased yield under treatment, the one‐tailed test focused specifically on the direction “control < treated” resulted in a lower *p* value (*p* = 0.184). Although still above the conventional *α* = 0.05 threshold, the directional test indicates a coherent trend toward higher productivity in the treated areas. This interpretation is further supported by the effect size metrics: Cliff's Delta (*δ* = 0.244) reveals a small but positive shift in the distribution, meaning treated samples more frequently yield higher values than controls. The bootstrap 95% interval (−0.266 to 0.733) reflects uncertainty typical of modest sample sizes, yet still centers on a positive effect. Likewise, the Vargha & Delaney A measure (*A*₁₂ = 0.622; 95% CI: 0.367 to 0.867) indicates that a randomly selected treated observation has approximately a 62% probability of exceeding a control observation. Together, these directional effect sizes reinforce the presence of a consistent tendency toward increased productivity under the treatment, even in the absence of conventional statistical significance.

**TABLE 2:** Results of the Mann–Whitney *U* test comparing soybean yield between control and treated groups.

Mann–Whitney *U* test			
Alternative hypothesis	*U*	*p*	Significance (*p* < 0.05)
Control ≠ Treated	33.50	0.369	ns
Control < Treated	33.50	0.184	ns

*Note:* The table reports the *U* statistic, *p* values, and statistical significance for two alternative hypotheses (two‐tailed and one‐tailed). ‘ns’ indicates no statistically significant difference between groups (*p* ≥ 0.05).

The greatest proportional gain related to the treatment was observed in Camaquã, where productivity increased from 89.6 to 95.5 bags per hectare, resulting in a 7% improvement (see Table 1). These absolute values remain far above both the municipal average yield of 45.7 bags per hectare and the statewide mean of 43.4 bags per hectare, indicating that the treatment enhanced productivity under already favourable baseline conditions (EMATER/RS 2025a, 2025b).

In Dom Pedrito and Candelária, the treatment also promoted a 6% increase in yield in both municipalities. In Dom Pedrito, productivity rose from 50.3 to 53.2 bags per hectare, clearly exceeding the local average of 30.2 bags per hectare and the statewide mean. In Candelária, productivity increased from 52.6 to 55.6 bags per hectare, surpassing the municipal average of 50.3 bags per hectare (EMATER/RS 2025b). Although the magnitude of improvement in these municipalities was smaller than that observed in Camaquã, the treatment‐driven increases still represent meaningful positive responses relative to the typical yield levels of each region.

Cumulative precipitation throughout the soybean growing cycle (November to May) was directly proportional to plant development and final productivity at each location. Camaquã, with the highest productivity, recorded the highest rainfall (652.6 mm), followed by Candelária (560.2 mm) and Dom Pedrito (536.8 mm), suggesting an important interaction between water availability and treatment effectiveness. Pearson's correlation analysis revealed a strong positive association between rainfall and yield (*r* = 0.99). Under the one‐tailed hypothesis that higher rainfall increases productivity, the test yielded *t* = 7.05 and a corresponding *p* value of 0.0449.

### 3.2 Soil Physicochemical and Enzymatic Stability Across Control and Treated Areas

Results of the physicochemical and enzymatic analyses comparing control and treated areas, aggregated across all sampling locations and time points (*n* = 12 per treatment), revealed no statistically significant differences for any of the evaluated variables. Using the Mann–Whitney *U* test with a significance threshold of *p* < 0.05, no parameter displayed evidence of treatment‐induced effects under the conditions assessed. These findings indicate that the treatment did not produce a detectable impact on soil physicochemical or enzymatic profiles, suggesting stability of these indicators irrespective of treatment application. The corresponding descriptive and inferential statistics (mean, SD, SE, coefficient of variation, *U*, and *p* values) are presented in the Table S2, and the complete soil physical, chemical, and biological dataset is provided in the Supporting Information (Soil_Analysis_Database_Physical_Chemical_Biological_Parameters.xlsx).

### 3.3 Treatment Consistently Enhances Vegetative Growth, With Strongest Effects on Shoot and Root Biomass

Across the three evaluated locations, the treatment affected plant development with distinct magnitudes and temporal patterns, but a common trend of enhanced vegetative growth was observed, particularly for biomass‐related traits. The effects were evaluated separately for each city and sampling time by comparing control and treated plants using non‐parametric statistics, allowing the quantification of treatment‐induced changes through both percentage increases and significance testing. A detailed summary of treatment effects across locations and sampling times is presented in Table 3, while complete descriptive statistics for all evaluated variables are provided in the Table S3.

**TABLE 3:** Statistical analysis of plant growth parameters measured in control and treated soybean plants across locations (Dom Pedrito, Candelária, and Camaquã) and sampling periods (Sampling 1 and 2).

Location	Sampling	Statistical analysis	Plant stand	Shoot length (cm)	Root length (cm)	Shoot biomass (g)	Root biomass (g)
Dom Pedrito	1	Control	10.9	12.8	10.3	—	—
		Treated	11.1	13.3	10.9	—	—
		Treatment effect (%)	2%	4%	6%	—	—
		Significance	ns	ns	ns	—	—
		*p*	0.847	0.478	0.468	—	—
		Rank‐biserial correlation	0.060	−0.190	−0.200	—	—
		SE Rank‐biserial correlation	0.259	0.259	0.259	—	—
	2	Control	10.9	25.1	14.5	28.3	7.85
		Treated	11.1	34.6	16.5	42.1	10.6
		Treatment effect (%)	2%	38%	14%	49%	35%
		Significance	ns	***	ns	***	***
		*p*	0.847	< 0.001	0.069	< 0.001	< 0.001
		Rank‐biserial correlation	0.060	−1.000	−0.480	−1.000	−1.000
		SE Rank‐biserial correlation	0.259	0.259	0.259	0.259	0.259
Candelária	1	Control	10.8	15.9	11.8	9.95	2.4
		Treated	12	20.4	14	13.65	3.1
		Treatment effect (%)	11%	28%	19%	37%	29%
		Significance	ns	**	**	***	**
		*p*	0.073	0.004	0.005	< 0.001	0.002
		Rank‐biserial correlation	−0.47	−0.77	−0.74	−1	−0.75
		SE Rank‐biserial correlation	0.259	0.259	0.259	0.259	0.259
	2	Control	10.8	34	19.9	66.3	13.05
		Treated	12	40.5	24.6	90.95	17.7
		Treatment effect (%)	11%	19%	24%	37%	36%
		Significance	ns	***	*	***	***
		*p*	0.073	< 0.001	0.034	< 0.001	< 0.001
		Rank‐biserial correlation	−0.470	−0.940	−0.570	−1.000	−1.000
		SE Rank‐biserial correlation	0.259	0.259	0.259	0.259	0.259
Camaquã	1	Control	10.1	22.1	11.5	25.3	4.8
		Treated	11.7	26.5	13.5	41.25	8.8
		Treatment effect (%)	16%	20%	17%	63%	83%
		Significance	*	**	*	***	***
		*p*	0.033	0.002	0.041	< 0.001	< 0.001
		Rank‐biserial correlation	−0.560	−0.830	−0.540	−1.000	−1.000
		SE Rank‐biserial correlation	0.259	0.259	0.259	0.259	0.259
	2	Control	10.1	33.8	16.7	75.35	21.35
		Treated	11.7	43.1	23.2	137.9	30.45
		Treatment effect (%)	16%	28%	39%	83%	43%
		Significance	*	***	***	***	***
		*p*	0.033	< 0.001	< 0.001	< 0.001	< 0.001
		Rank‐biserial correlation	−0.560	−1.000	−0.940	−1.000	−1.000
		SE Rank‐biserial correlation	0.259	0.259	0.259	0.259	0.259

*Note:* The table reports mean values for plant stand, shoot length, root length, shoot biomass, and root biomass for control and treated groups, followed by the relative treatment effect (%). Statistical significance was assessed using the Mann–Whitney *U* test, with corresponding *p* values and significance levels, where * indicates *p* < 0.05, ** indicates *p* < 0.01, and *** indicates *p* < 0.001; ns denotes no statistically significant difference. Effect size is expressed as rank‐biserial correlation, with its associated standard error (SE). Missing values (−) indicate parameters not evaluated in a given sampling.

In Dom Pedrito, no statistically significant differences between control and treated plants were detected at the first sampling for plant stand, shoot length, or root length, with low percentage increases ranging from 2% to 6% (*p* > 0.05). At the second sampling, however, clear treatment effects emerged. Shoot length increased by 38% in treated plants compared to the control (*p* < 0.001), while shoot and root biomass increased by 49% and 35%, respectively (both *p* < 0.001). Root length showed a 14% increase, but this difference was not statistically significant (*p* = 0.069). Plant stand remained unaffected by the treatment at both sampling times.

In Candelária, plant stand did not differ significantly between treatments in either sampling, despite a consistent numerical increase of 11% (*p* = 0.073). In contrast, growth and biomass variables responded significantly to the treatment. At the first sampling, shoot length and root length increased by 28% (*p* = 0.004) and 19% (*p* = 0.005), respectively, while shoot biomass increased by 37% (*p* < 0.001) and root biomass by 29% (*p* = 0.002). These effects persisted at the second sampling, with increases of 19% for shoot length (*p* < 0.001), 24% for root length (*p* = 0.034), 37% for shoot biomass (*p* < 0.001), and 36% for root biomass (*p* < 0.001).

The most pronounced and consistent treatment effects were observed in Camaquã. At the first sampling, all evaluated variables differed significantly between control and treated plants. Plant stand increased by 16% (*p* = 0.033), shoot length by 20% (*p* = 0.002), and root length by 17% (*p* = 0.041). Substantially larger increases were detected for biomass variables, with shoot biomass increasing by 63% and root biomass by 83% relative to the control (both *p* < 0.001). At the second sampling, treatment effects intensified further, with increases of 28% in shoot length, 39% in root length, 83% in shoot biomass, and 43% in root biomass (all *p* < 0.001), while the increase in plant stand remained significant at 16% (*p* = 0.033).

Overall, the treatment consistently promoted increases in shoot and root biomass across all locations and sampling times in which significant effects were detected, whereas effects on plant stand were location‐dependent. The magnitude of the response varied among cities and between sampling times, with the largest percentage increases and strongest statistical support generally observed for biomass‐related variables.

### 3.4 Limited Suppression of Phytoparasitic Nematodes by *Bacillus firmus* –Based Treatment Under Field Conditions

**FIGURE 3:**
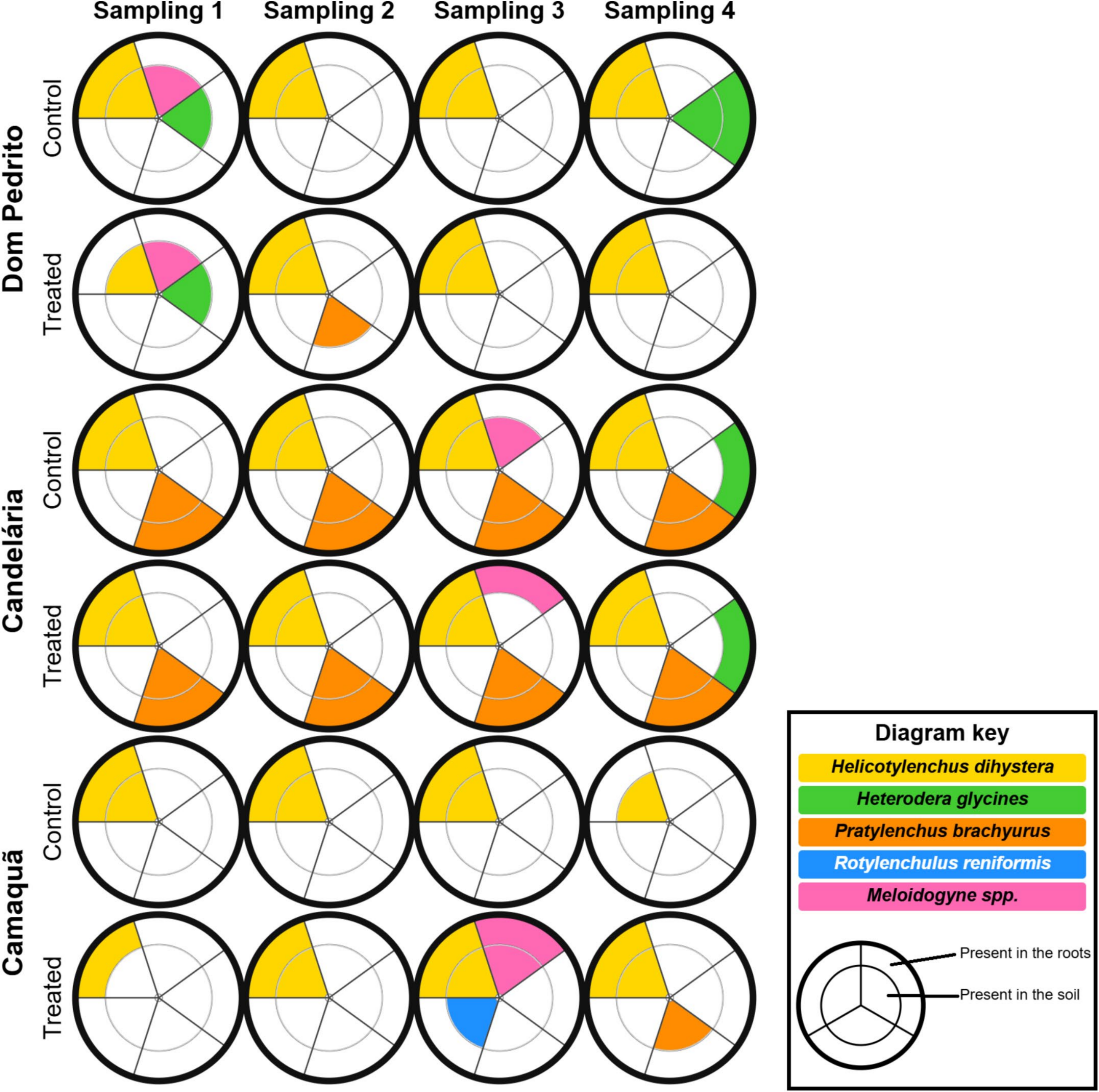
Distribution of plant‐parasitic nematodes across sampling periods and treatments in the analysed municipalities. Each circular diagram represents one sample and is divided into five triangular sectors corresponding to the nematode species or genus indicated on the outer ring. Each sector is further subdivided into two concentric regions, representing nematode presence in plant roots (outer region) and soil (inner region). Coloured regions indicate presence, whereas white regions indicate absence. Samples are grouped by treatment (Control and Treated) and sampling period for each municipality. The diagram key summarises the colour coding and the graphical structure used to interpret the diagrams.

Molecular analysis revealed complex dynamics of phytoparasitic nematode presence and distribution across experimental sites, treatments, and sampling periods. Overall, results demonstrated the persistence of economically important nematode species despite seed treatment with the *Bacillus* *firmus*‐based bionematicide, suggesting limitations in the biocontrol agent's effectiveness under field conditions (Figure 3).

In Dom Pedrito, *Helicotylenchus dihystera* was consistently detected in both soil and roots of all samples (control and treated) across samplings, with the exception of treated samples during sampling 1 (S1), where *H. dihystera* was found only in soil. In S1, besides *H. dihystera*, *Meloidogyne* spp. and *Heterodera glycines* were detected in soil from both control and treated samples. In sampling 4 (S4), *H. glycines* was identified in both soil and roots of control samples, while sampling 2 showed *Pratylenchus brachyurus* ' presence in soil from treated areas.

In Candelária, *H. dihystera* and *P. brachyurus* were consistently detected in both soil and roots, in control and treated areas, during all four samplings. sampling 3 additionally revealed *Meloidogyne* spp. in soil from control areas and roots of treated areas. In S4, *H. glycines* was also recorded in roots from both control and treated areas, emphasising the complexity of nematode communities in this region.

In Camaquã, *H. dihystera* was detected in soil and roots of control samples in samplings 1, 2, and 3, but only in soil during S4. In treated samples, S1 showed *H. dihystera* exclusively in roots, whereas sampling 2 revealed its presence in both soil and roots. sampling 3 exhibited greater species diversity in treated areas, with *H. dihystera* and *Meloidogyne* spp. in both soil and roots, plus *Rotylenchulus reniformis* in soil. S4 showed *H. dihystera* in both soil and roots of treated areas, along with *P. brachyurus* in soil.

These findings reveal the continuous presence of *H. dihystera* across samples, with variations in species detected and their distribution across samplings and areas, suggesting complex interactions between nematode populations, soil environment, and applied treatments.

### 3.5 Site‐ and Time‐Dependent Shifts in Bacterial and Fungal Genera Show Limited Treatment Effects

**FIGURE 4:**
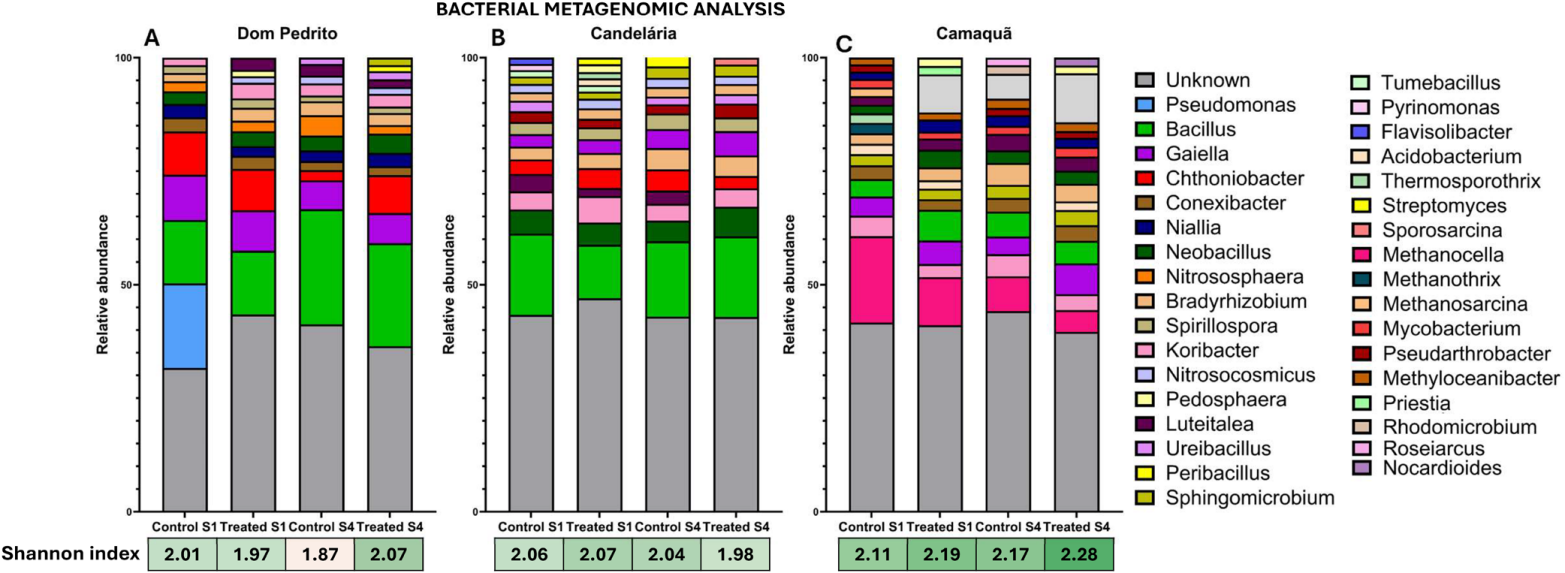
Relative abundance of bacterial genera identified in soil samples from three locations: Dom Pedrito, Candelária, and Camaquã. Each bar represents a sample classified by treatment and sampling point: “Control S1”, “Control S4”, “Treated S1”, and “Treated S4”. The *y*‐axis represents the relative abundance (%) of each bacterial genus. Colours in the stacked bars correspond to different bacterial genera, as indicated in the legend on the right. Genera labelled as “Unknown” refer to sequences that could not be taxonomically classified at the genus level. The diversity of bacterial communities was assessed using the Shannon diversity index, shown below each group of bars.

The relative abundance of bacterial genera and the Shannon diversity index across treatments, locations, and sampling times are shown in Figure 4. In the stacked bar plots, each colour represents a distinct bacterial genus, with colours kept consistent across samples to facilitate visual comparison of community composition. Across all locations and sampling times, the bacterial communities were dominated by the genera *Bacillus*, *Gaiella*, *Chthoniobacter*, *Conexibacter*, and *Bradyrhizobium*, together with a substantial proportion of sequences classified as “Unknown.” Genera labelled as “Unknown” correspond to sequences that could not be taxonomically assigned at the genus level and were retained as a separate category in the calculation of relative abundance, without genus‐level ecological interpretation. An integrated evaluation of Figure 4 and the relative abundance values provided in the Table S4 indicated that no bacterial genus exhibited a consistent modulation across all sampling times and locations in response to the treatment. This conclusion is supported by the statistical analysis performed on the dominant genera (Table S5), which revealed no statistically significant differences attributable to the treatment.

Consistent with the lack of a treatment effect observed for the dominant genera as a whole, the genus *Bacillus* also showed no statistically significant differences in relative abundance between treated and control samples (see Table S5). When all locations were analysed jointly, the Wilcoxon signed‐rank test revealed no significant treatment effect at either Sampling 1 (*p* = 1.00) or Sampling 4 (*p* = 0.50). Although the effect size at Sampling 4 indicated a moderate rank‐biserial correlation (*r* = 0.667), this pattern did not reach statistical significance, suggesting that the observed variation in *Bacillus* abundance was not consistently associated with the treatment. However, the small sample size limits statistical power.

Despite the absence of an overall treatment effect, descriptive analyses revealed location‐ and time‐dependent variation in *Bacillus* relative abundance (Figure 4; Table S4). In Dom Pedrito (see Figure 4A), similar abundances were observed between control (13.9%) and treated samples (14.1%) at Sampling 1, followed by an increase in both conditions at Sampling 4 (25.4% in the control and 22.7% in treated samples). In Candelária (see Figure 4B), *Bacillus* abundance was lower in treated samples (11.8%) than in the control (17.8%) at Sampling 1, whereas comparable values were observed at Sampling 4 (16.6% in the control and 17.7% in treated samples). In Camaquã (see Figure 4C), overall *Bacillus* abundance was lower than in the other locations; treated samples showed higher values at Sampling 1 (6.7%) compared to the control (3.8%), while similar abundances were detected at Sampling 4 (5.4% and 5.0%, respectively).

**FIGURE 5:**
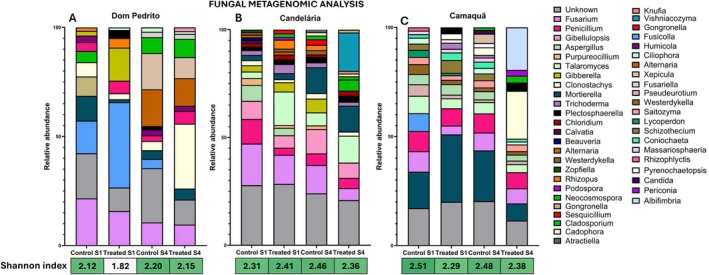
Relative abundance of fungal genera identified in soil samples from three locations: Dom Pedrito, Candelária, and Camaquã. Each bar represents a sample categorised by treatment and sampling point: “Control S1”, “Control S4”, “Treated S1”, and “Treated S4”. The y‐axis indicates the relative abundance (%) of each fungal genus. Different colours in the stacked bars correspond to different fungal genera, as shown in the legend on the right side of the figure. The category “Unknown” includes sequences that could not be classified at the genus level. Fungal community diversity is represented by the Shannon diversity index displayed below each set of bars.

Across the three municipalities, fungal communities exhibited pronounced temporal dynamics and location‐specific responses to treatment (see Figure 5 and Table S6). In Dom Pedrito, treated samples showed marked shifts in community composition between sampling events, including the strong increase of *Clonostachys* in Sampling 4 (Figure 5A). In Candelária, treatment‐associated responses were more genus‐specific, with taxa such as *Vishniacozyma* becoming prominent exclusively in treated samples at Sampling 4 (Figure 5B), while remaining absent in the other locations. In Camaquã, a distinct pattern was observed, with treated samples at Sampling 4 characterised by the emergence of *Albifimbria*, a genus not detected in earlier sampling events (Figure 5C). Temporal succession was evident across all municipalities, as several genera were detected exclusively at Sampling 4, indicating time‐dependent community restructuring rather than a uniform treatment effect. Despite these site‐specific and temporal shifts, statistical analyses performed on the dominant genera did not reveal significant treatment‐associated effects (data not shown), reinforcing the conclusion that fungal community responses were primarily shaped by local environmental conditions.

### 3.6 Soil Microbial Diversity Driven Primarily by Temporal Progression, With Limited Treatment Influence

#### 3.6.1 Alpha Diversity (α) Analysis

Alpha diversity was evaluated using the Shannon index to compare microbial diversity between control and treated samples at two sampling times (S1 and S4). Paired comparisons were performed using the Wilcoxon signed‐rank test, pooling data across locations to ensure sufficient sample size. Effect sizes were estimated using the rank‐biserial correlation. Complete statistical results, including *p* values and effect size estimates for all comparisons, are provided in Table S7.

For bacterial communities, no statistically significant differences in Shannon diversity were detected between control and treated samples at either sampling time (S1: *p* = 0.75; S4: *p* = 0.586). In S1, the effect size indicated a small‐to‐moderate asymmetry in paired comparisons (rank‐biserial correlation = 0.333), reflecting modest variation in Shannon values between treatments across locations. In S4, a moderate negative rank‐biserial correlation was observed (−0.5), corresponding to higher mean Shannon index values in treated samples compared to controls, although this pattern was not statistically significant.

For fungal communities, no statistically significant differences were detected between treatments in either sampling (S1: *p* = 0.5; S4: *p* = 0.5). Effect size estimates showed moderate‐to‐large negative rank‐biserial correlations at both time points (−0.667 in S1 and S4), indicating that mean Shannon diversity values were higher in treated samples than in controls across pooled locations. Despite this directional pattern, the observed differences were not sufficient to reach statistical significance.

Overall, the alpha diversity analysis indicates that the treatment did not significantly affect Shannon diversity of bacterial or fungal communities at either sampling time. Nonetheless, effect size estimates revealed asymmetries in paired comparisons, particularly for fungal communities and for bacterial communities at the later sampling, corresponding to higher mean diversity values in treated samples. These patterns suggest heterogeneous responses across locations and time points rather than a uniform treatment effect.

#### 3.6.2 Beta Diversity (β) Analysis of Soil Bacterial Communities

**FIGURE 6:**
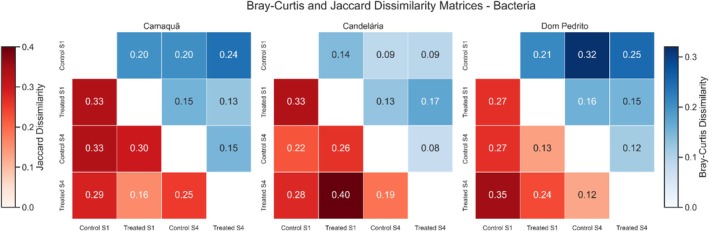
Dissimilarity matrices calculated using the Bray‐Curtis (upper diagonal, blue scale) and Jaccard (lower diagonal, red scale) indices for bacterial communities in soil samples from three locations: Camaquã, Candelária, and Dom Pedrito. The *x*‐ and *y*‐axes represent the sample IDs and treatments: “Control S1”, “Treated S1”, “Control S4”, and “Treated S4”. Each cell indicates the dissimilarity between a pair of samples, ranging from 0 (highest similarity) to 1 (highest dissimilarity). Colour bars on the left and right represent the Jaccard (red) and Bray‐Curtis (blue) scales, respectively. Sample order is identical across matrices to facilitate comparison between indices and locations.

Beta diversity patterns based on Bray–Curtis dissimilarity revealed clear effects of sampling time (phenological stage) and more context‐dependent effects of the treatment on soil bacterial community structure throughout the soybean cycle. Bray–Curtis dissimilarity values reflect changes in the relative abundance of taxa between samples, allowing the evaluation of both temporal dynamics and treatment‐related shifts in microbial composition at the R2 (full flowering–S1) and R7 (beginning of maturity–S4) stages (see Figure 6).

**FIGURE 7:**
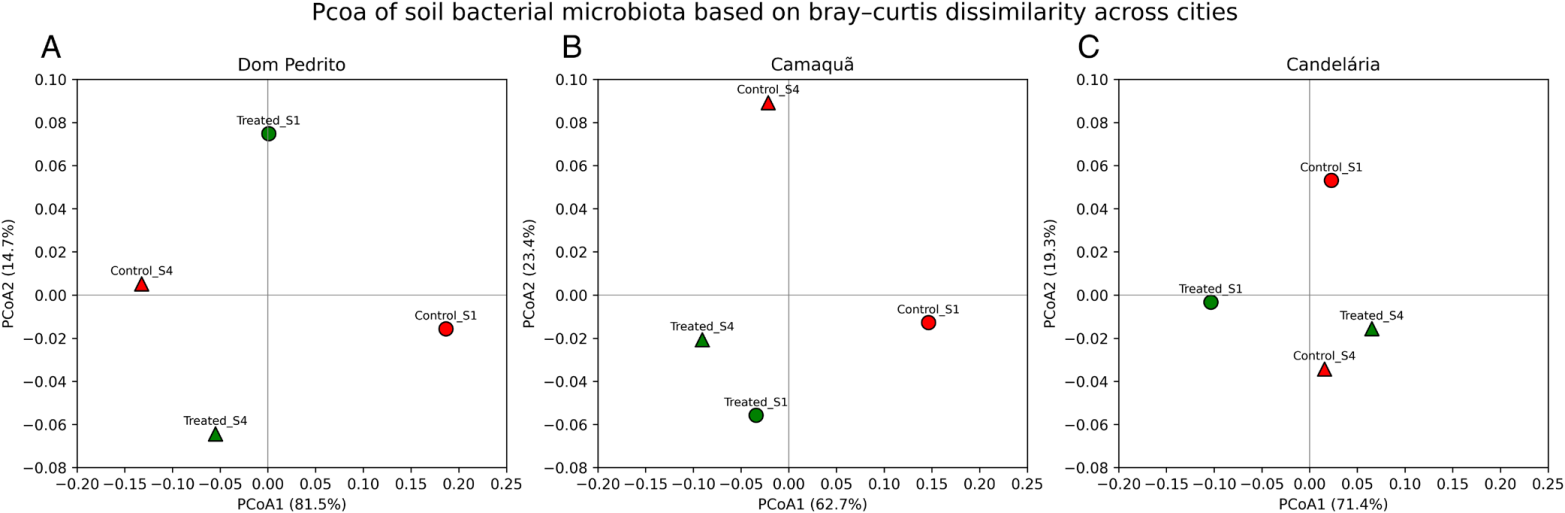
Principal coordinates analysis (PCoA) of soil bacterial microbiota based on Bray–Curtis dissimilarity, performed separately for each city: Dom Pedrito (A), Camaquã (B), and Candelária (C). Each panel represents an independent ordination space calculated using the Bray–Curtis distance matrix of bacterial community composition for the respective location. Points correspond to individual samples, identified by treatment (control or treated) and sampling time (S1 or S4), as indicated by the sample labels. Samples from Sampling 1 are represented by circles, whereas samples from Sampling 4 are represented by triangles. Red‐filled symbols indicate control samples, while green‐filled symbols indicate treated samples. The first (PCoA1) and second (PCoA2) principal coordinate axes are shown on the x‐ and y‐axes, respectively, with the percentage of explained variance indicated in parentheses for each axis. Differences in the spatial distribution of samples reflect variation in bacterial community structure associated with treatment and sampling time within each city. The origin (0, 0) is marked by intersecting reference lines to aid visual interpretation of sample dispersion and relative dissimilarities.

Across the three evaluated locations, ordination analyses (PCoA) based on Bray–Curtis distances revealed clear temporal and treatment‐related structuring of bacterial communities, with the relative importance of these factors varying among sites (see Figure 7). PERMANOVA analyses confirmed that sampling time (S1 vs. S4) explained a substantial proportion of the variance in Dom Pedrito (*R* ^2^ = 0.24, *p* = 0.010) and Candelária (*R* ^2^ = 0.19, *p* = 0.030), exceeding the contribution of the treatment effect in these locations (*R* ^2^ = 0.13, *p* = 0.040; and *R* ^2^ = 0.14, *p* = 0.050, respectively). In contrast, at Camaquã, treatment application emerged as the primary driver of bacterial community differentiation (*R* ^2^ = 0.21, *p* = 0.020), surpassing the effect of sampling time (*R* ^2^ = 0.16, *p* = 0.040). The complete PERMANOVA results, including *R* ^2^ values and statistical significance for all tested factors, are provided in Table S8.

Collectively, these results indicate that temporal succession during soybean development was a major driver of bacterial community turnover in most locations, while the magnitude and dominance of the treatment effect were strongly site‐dependent, reflecting the influence of local environmental conditions on microbial community responses.

In Dom Pedrito, Bray–Curtis dissimilarities indicated pronounced temporal changes in bacterial community composition. The dissimilarity between Control S1 and Control S4 (0.32) demonstrates substantial community restructuring over the crop cycle, even without treatment. Similarly, Treated S1 versus Treated S4 dissimilarity was relatively high (0.41), indicating strong temporal succession under treated conditions. PCoA ordination revealed clearer separation between S1 and S4 samples than between control and treated samples (Figure 7). PERMANOVA confirmed this pattern, with sampling time explaining a larger proportion of variance (*R* ^2^ = 0.24, *p* = 0.010) compared to treatment (*R* ^2^ = 0.13, *p* = 0.040). These results indicate that bacterial community dynamics were primarily driven by temporal factors associated with plant development and soil–plant interactions, while treatment exerted a smaller, secondary influence.

In Candelária, Bray–Curtis dissimilarities were generally lower, reflecting greater overall community stability. Dissimilarities between Control S1 and S4 (0.09) and Treated S1 and S4 (0.17) indicate limited temporal turnover relative to Dom Pedrito. PCoA showed tighter clustering of samples, with modest separation along both temporal and treatment gradients. PERMANOVA results revealed that sampling time (*R* ^2^ = 0.19, *p* = 0.030) explained slightly more variance than treatment (*R* ^2^ = 0.14, *p* = 0.050), though both factors were significant. These findings suggest that, in Candelária, the soil bacterial community was comparatively stable over time, and the treatment effect, while detectable, did not strongly alter community structure.

Camaquã exhibited an intermediate but distinct pattern. Bray–Curtis dissimilarities indicated moderate differences between control and treated samples at S1, followed by reduced dissimilarity at S4, suggesting partial convergence over time. PCoA ordination showed contributions from both treatment and sampling time. PERMANOVA indicated that treatment explained a slightly larger proportion of variance (*R* ^2^ = 0.21, *p* = 0.020) than sampling time (*R* ^2^ = 0.16, *p* = 0.040), contrasting with the other locations. These results suggest that, in Camaquã, the bacterial community was more responsive to the treatment than to temporal progression alone, highlighting the influence of local environmental or edaphic conditions on microbial responses.

Bray–Curtis‐based analyses highlight changes in relative abundances of bacterial taxa, revealing that both sampling time and treatment influenced community structure, with temporal effects dominant in most locations except Camaquã. Complementary inspection of Jaccard dissimilarities, which consider presence–absence data, confirmed that qualitative shifts in composition also occurred. In several comparisons, Jaccard values were slightly higher than Bray–Curtis, indicating that changes involved both abundance redistribution and gain/loss of taxa. Both indices consistently showed stronger differentiation at S1, with differences between control and treated samples tending to decrease by S4, reflecting community stabilisation over time.

Overall, the combined *Bacillus firmus* and fungicide treatment induced detectable but context‐dependent shifts in soil bacterial communities. Temporal succession during soybean development was the main driver of beta diversity patterns, while treatment acted as a secondary, modulating factor, with effects varying among locations.

#### 3.6.3 Beta Diversity (β) Analysis of Fungal Communities

**FIGURE 8:**
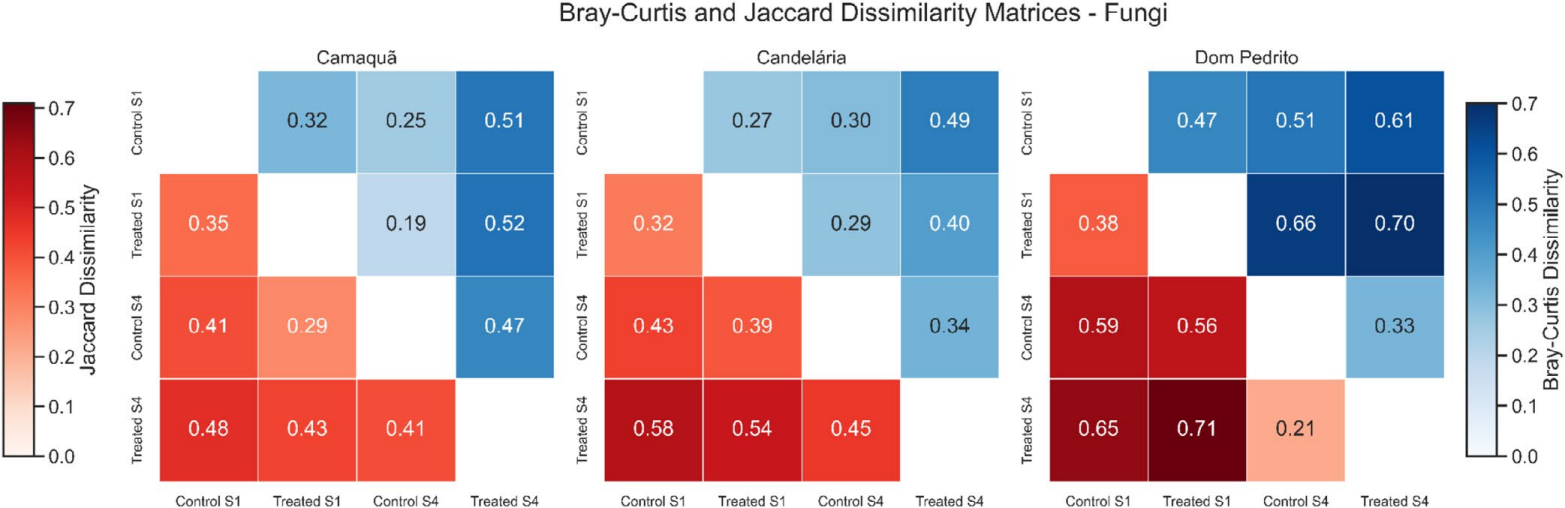
Dissimilarity matrices calculated using the Bray‐Curtis (upper diagonal, blue scale) and Jaccard (lower diagonal, red scale) indices for fungal communities in soil samples from three locations: Camaquã, Candelária, and Dom Pedrito. The *x*‐ and y‐axes represent the sample IDs and treatments: “Control S1”, “Treated S1”, “Control S4”, and “Treated S4”. Each cell indicates the dissimilarity between a pair of samples, ranging from 0 (highest similarity) to 1 (highest dissimilarity). Colour bars on the left and right represent the Jaccard (red) and Bray‐Curtis (blue) scales, respectively. Sample order is identical across matrices to facilitate comparison between indices and locations.

Beta diversity analyses based on Bray–Curtis and Jaccard dissimilarity matrices clearly demonstrated that soil fungal communities were not identical among the compared samples, indicating measurable variation in microbiota composition across space and time (see Figure 8). Across all evaluated locations, dissimilarity values revealed real differences between sample pairs, reflecting both shifts in the relative abundance of dominant taxa and qualitative changes in species presence–absence. These results confirm that fungal communities exhibit intrinsic heterogeneity and dynamic behaviour, which is expected for microbial assemblages under field conditions.

**FIGURE 9:**
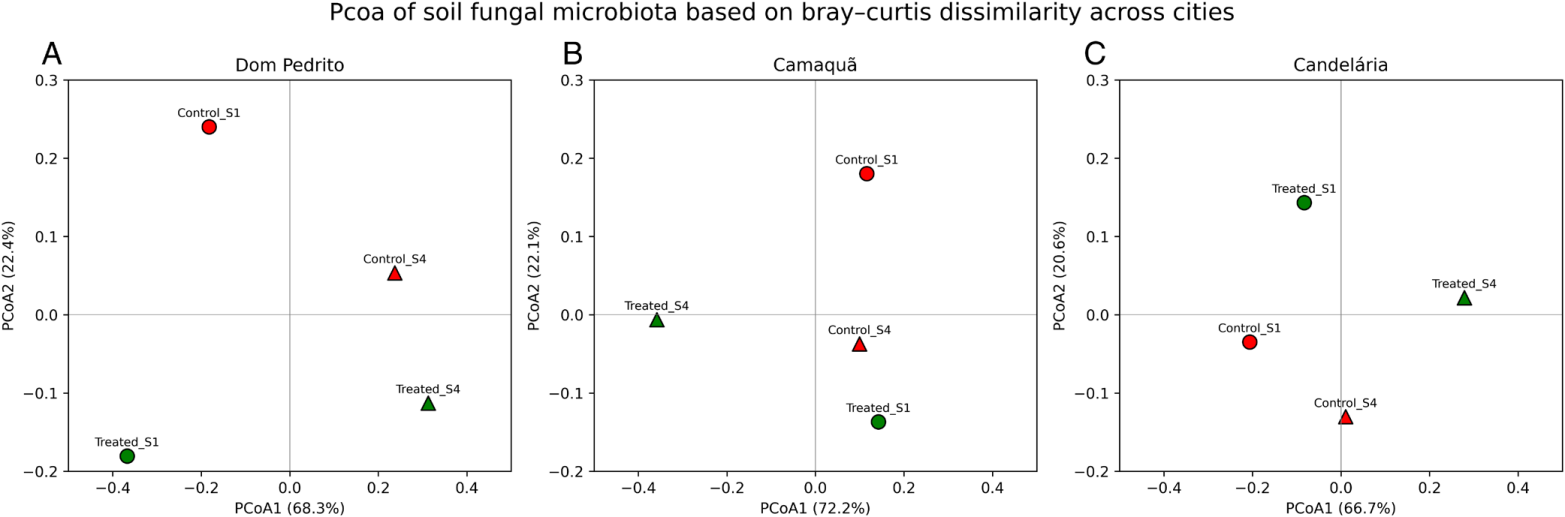
Principal coordinates analysis (PCoA) of soil fungal microbiota based on Bray–Curtis dissimilarity, performed separately for each city: Dom Pedrito (A), Camaquã (B), and Candelária (C). Each panel represents an independent ordination space calculated using the Bray–Curtis distance matrix of bacterial community composition for the respective location. Points correspond to individual samples, identified by treatment (control or treated) and sampling time (S1 or S4), as indicated by the sample labels. Samples from Sampling 1 are represented by circles, whereas samples from Sampling 4 are represented by triangles. Red‐filled symbols indicate control samples, while green‐filled symbols indicate treated samples. The first (PCoA1) and second (PCoA2) principal coordinate axes are shown on the *x*‐ and *y*‐axes, respectively, with the percentage of explained variance indicated in parentheses for each axis. Differences in the spatial distribution of samples reflect variation in bacterial community structure associated with treatment and sampling time within each city. The origin (0, 0) is marked by intersecting reference lines to aid visual interpretation of sample dispersion and relative dissimilarities.

However, ordination patterns derived from Principal Coordinates Analysis (PCoA) did not show a consistent separation of samples according to treatment application or sampling time (see Figure 9). Although specific pairwise comparisons exhibited moderate to high dissimilarity values, the spatial distribution of samples in the multivariate ordination space did not follow a clear directional gradient associated with the tested experimental factors. Thus, while the dissimilarity matrices capture genuine differences among communities, they do not, by themselves, indicate a systematic effect of treatment or temporal progression.

In Camaquã, comparisons between Control S1 and Treated S4 samples showed moderate dissimilarity (Bray–Curtis = 0.51; Jaccard = 0.48), indicating compositional changes over the experimental period. Nevertheless, PERMANOVA results demonstrated that neither treatment (*R* ^2^ = 0.43, *p* = 0.354) nor sampling time (*R* ^2^ = 0.37, *p* = 0.689) explained these differences in a statistically consistent manner (Table S8). Despite relatively high proportions of explained variance, the lack of statistical significance indicates that the observed variability cannot be robustly attributed to the tested factors and is more consistent with natural community fluctuations.

In Dom Pedrito, the highest dissimilarity values were observed between Treated S1 and Treated S4 samples (Bray–Curtis = 0.70; Jaccard = 0.71), revealing pronounced temporal variation in fungal community composition. However, PERMANOVA did not detect a significant effect of sampling time (*R* ^2^ = 0.57, *p* = 0.339) or treatment (*R* ^2^ = 0.08, *p* = 1.000). These results indicate that, although dissimilarity matrices capture clear differences among samples, such differences were not sufficiently consistent across replicates to be attributed to a temporal pattern or treatment effect.

In Candelária, the largest divergences were also observed between Control S1 and Treated S4 samples (Bray–Curtis = 0.49; Jaccard = 0.58), with slightly higher Jaccard values suggesting a greater contribution of qualitative taxonomic changes. Nonetheless, PERMANOVA analyses again revealed no significant effects of treatment (*R* ^2^ = 0.16, *p* = 1.000) or sampling time (*R* ^2^ = 0.41, *p* = 0.651). A substantial fraction of the variation remained associated with the residual component (*R* ^2^ = 0.43), highlighting pronounced intrinsic heterogeneity in fungal communities at this site.

Overall, the dissimilarity matrices unequivocally demonstrate that soil fungal communities differ among samples when compared pairwise. However, PERMANOVA results indicate that these differences do not follow a consistent pattern that can be attributed to treatment application or sampling time. Consequently, the observed variation is best interpreted as arising from local ecological processes, environmental heterogeneity, and the inherent dynamics of microbial communities, rather than from directional effects induced by the experimental conditions.

Across all locations, beta diversity analyses revealed contrasting patterns for bacterial and fungal communities. Soil bacterial communities showed clear temporal structuring, with significant contributions of both sampling time and treatment (PERMANOVA; see Table S8), although the relative importance of these factors varied among sites. In contrast, fungal communities, despite measurable pairwise dissimilarities, exhibited no statistically consistent effect of sampling time or treatment, indicating that observed variation primarily reflects intrinsic heterogeneity and local ecological factors. These results indicate that bacterial communities are generally more responsive to temporal and treatment‐related influences, whereas fungal communities maintain higher intrinsic variability under field conditions.

## 4 Discussion

### 4.1 Biomass Gains as the Primary Agronomic Response to the *Bacillus firmus* –Based Treatment

The combined seed treatment based on *Bacillus firmus* consistently enhanced vegetative performance, with biomass‐related traits emerging as the most robust and reproducible response across locations. As shown in Section 3.3, increases in shoot and root biomass were stronger and more statistically supported than effects on plant stand or linear growth, indicating that the treatment primarily improved physiological efficiency rather than uniformly stimulating all growth parameters.

Growth responses varied among locations and sampling periods, reinforcing the role of environmental context. Biomass gains were modest at early stages in Dom Pedrito but intensified later, whereas in Candelária and especially Camaquã, treatment effects were evident earlier and remained consistent. This temporal pattern suggests a cumulative effect that becomes more pronounced as plants establish functional root systems and interact with the modified rhizosphere, consistent with previous field studies reporting delayed yet sustained growth promotion by *B. firmus* I‐1582 (Ghahremani et al. 2020; Susic et al. 2020).

The predominance of biomass accumulation over linear growth aligns with established mechanisms of plant growth–promoting rhizobacteria (PGPR), particularly *Bacillus* spp., which often enhance dry matter production through improved nutrient uptake, carbon assimilation, and resource allocation rather than plant height (Figueiredo et al. 2008; Singh et al. 2024). These effects are commonly associated with phytohormone production, phosphate solubilisation, and improved mineral availability in the rhizosphere (Mendis et al. 2018; Sahu et al. 2018; Susic et al. 2020).

Indirect mechanisms may also have contributed to the observed responses. Volatile organic compounds produced by *Bacillus*, such as acetoin and 2,3‐butanediol, are known to stimulate root development and biomass accumulation. Although VOCs were not directly measured, the pronounced shoot and root biomass increases are consistent with this mode of action (Hofmann 2013; Poulaki and Tjamos 2023; Tahir et al. 2017; Wu et al. 2019).

The observed vegetative gains provide a mechanistic explanation for the consistent, albeit non‐significant, increases in soybean productivity reported in Section 3.1. Locations exhibiting the strongest biomass responses, particularly Camaquã, also showed the largest proportional yield gains, reinforcing the link between early vegetative vigour, enhanced photosynthetic capacity, and yield potential. This relationship is well documented in soybean, as higher seed and early plant vigour promote faster canopy establishment, greater leaf area development, and increased carbon assimilation, ultimately contributing to improved grain yield (Ebone et al. 2020).

Environmental conditions, especially water availability, strongly modulated treatment performance. The superior responses observed in Camaquã, which had the highest accumulated precipitation, are consistent with the known dependence of *Bacillus* activity and rhizosphere colonisation on soil moisture (Beeman and Tylka 2018; Beeman et al. 2019). Under adequate moisture, *B. firmus* may enhance plant growth even in the absence of marked nematode suppression, a pattern clearly reflected in the present results.

Overall, these findings indicate that under field conditions, the primary agronomic benefit of the combined seed treatment lies in growth promotion and biomass accumulation rather than direct phytoparasitic nematode control. The strong context dependence observed highlights the importance of integrating plant–microbe interactions with environmental drivers when interpreting the performance of *B. firmus* –based products throughout the crop cycle.

### 4.2 Limited Field‐Level Suppression of Phytoparasitic Nematodes and Implications for Biocontrol Efficacy

The results indicate that the seed treatment combining a *Bacillus firmus* –based bionematicide with chemical protection provided limited and inconsistent suppression of phytoparasitic nematodes under field conditions, with strong spatial and temporal variability. Although the treatment was designed to prevent root infestation by key target species— *Heterodera glycines* , *Meloidogyne* spp., *Rotylenchulus reniformis*, and *Pratylenchus brachyurus* —these nematodes were detected in treated plots throughout the crop cycle, indicating that population establishment was not effectively prevented.

In Candelária, the detection of *H. glycines* in roots at Sampling 4 and *Meloidogyne* spp. at Sampling 3 in treated plots indicates failures in root‐level suppression, while the persistent presence of *P. brachyurus* in both soil and roots across all samplings suggests that the treatment was largely ineffective against this species at this location. Similar persistence patterns have been reported for *P. brachyurus* under biological seed treatments, reflecting its migratory endoparasitic behaviour and high adaptability to different soil conditions (Beeman et al. 2019; Susic et al. 2020).

Across all locations, *Helicotylenchus dihystera* was consistently detected in both control and treated plots throughout the sampling periods, highlighting its resilience and confirming that the treatment had no measurable activity against this non‐target species. The widespread occurrence of *H. dihystera* corroborates previous surveys of Brazilian agricultural soils (Machado et al. 2019). Although historically considered of low economic relevance, recent studies indicate that high population densities may impair root development and reduce soybean productivity, reinforcing its agronomic relevance in integrated nematode management programs (Gomes et al. 2003; Machado et al. 2019).

An increase in nematode species diversity in later samplings, particularly in S3 and S4, further emphasizes the ecological complexity of the system and suggests that single‐application seed treatments are insufficient to maintain nematode suppression throughout the crop cycle. In Candelária and Camaquã, multiple target and non‐target species coexisted in treated plots, indicating that the treatment did not exert strong selective pressure capable of simplifying the nematode community. This pattern may reflect a reduction in dominance of specific species rather than effective population control and is consistent with the transient nature of biological seed treatments reported in the literature (Silva et al. 2018).

The persistence of phytoparasitic nematodes at levels comparable to control plots supports the interpretation that the treatment modulated population dynamics without achieving sustained suppression. This behaviour aligns with the proposed mode of action of *B. firmus* , which primarily interferes with nematode penetration and mobility through root surface colonisation and localised metabolite production rather than causing broad population collapse (Beeman and Tylka 2018; Xiong et al. 2015). Under field conditions, such mechanisms may reduce early infection pressure but are unlikely to prevent reinfestation over time.

Species‐specific variability in treatment response observed in this study is consistent with previous reports on *B. firmus* ‐based products (Beeman et al. 2019; Ghahremani et al. 2020; Susic et al. 2020). These findings reinforce the importance of context‐dependent nematode management, in which biological treatments are integrated with complementary strategies such as crop rotation, resistant cultivars, and cultural practices to mitigate the persistence of both target and non‐target species (Kratochvil et al. 2004; Timper 2014).

Environmental and agronomic factors likely contributed substantially to the observed variability in efficacy. Soil texture, moisture, pH, and organic matter content are known to influence the survival, mobility, and rhizosphere establishment of biocontrol agents, thereby modulating their performance (Vasantha Srinivasan et al. 2025). In addition, host plant susceptibility, formulation characteristics, application uniformity, and dosage further interact with local conditions, generating heterogeneous outcomes across locations and seasons (Bagale 2021; Brodeur 2012; Castillo et al. 2013; Silva et al. 2018).

Consistent with these observations, field studies evaluating *B. firmus* –based bionematicides against *H. glycines* have frequently reported no significant reductions in nematode reproduction, juvenile penetration, or root damage when compared to untreated controls (Beeman and Tylka 2018; Beeman et al. 2019). Similar inconsistencies have been documented for *Meloidogyne* spp., where biological nematicides often enhanced plant growth without producing proportional reductions in nematode populations, particularly under open‐field conditions (d'Errico et al. 2019; Ghahremani et al. 2020; Huang et al. 2021; Pontes 2022).

Although laboratory and greenhouse studies demonstrate that *B. firmus* I‐1582 possesses multiple nematicidal and nematostatic mechanisms—such as inhibition of egg hatching, reduced juvenile motility, root colonisation, and induction of systemic resistance—these effects are not consistently translated into effective field‐level control (Mendoza et al. 2008; Schrimsher 2013; Mendis et al. 2018; Zhao et al. 2023). Together, the present results reinforce the notion that the agronomic value of *B. firmus* –based treatments under field conditions lies more in indirect plant performance benefits than in reliable nematode suppression, underscoring the need for integrated and site‐specific management strategies.

### 4.3 Context‐Dependent Temporal Dynamics of Bacillus in Response to Seed Treatment

The dynamics of the genus *Bacillus* were primarily structured by temporal progression and site‐specific conditions, with no consistent or sustained effect of the seed treatment across locations. Although the treatment included a *Bacillus firmus* –based bionematicide, changes in *Bacillus* relative abundance were heterogeneous and transient, reinforcing the strong environmental control over this genus in agricultural soils (Delgado‐Baquerizo et al. 2016).

Across sites, early responses to the treatment were variable, including slight reductions, increases, or no change in *Bacillus* abundance, depending on location. However, these initial differences tended to dissipate by the later sampling, indicating convergence between treated and control plots over time. This pattern suggests that temporal factors related to crop development, seasonal dynamics, and soil conditions exerted a stronger influence on *Bacillus* abundance than the applied inputs, consistent with previous field studies highlighting time as a dominant driver of bacterial community dynamics (Lupwayi et al. 2019; Zhao et al. 2023).

The transient nature of treatment‐associated responses, particularly where short‐term increases in *Bacillus* abundance were observed, is consistent with limited persistence of introduced strains within resident soil microbiota. Competition with native populations and environmental filtering likely constrained long‐term establishment, a phenomenon widely reported for *Bacillus*‐based inoculants under field conditions (Beeman and Tylka 2018; Beeman et al. 2019; Susic et al. 2020).

The absence of statistically significant differences in *Bacillus* abundance between treated and control samples across all locations further supports the interpretation that the seed treatment modulated local and short‐term dynamics without overriding broader ecological processes. It is important to note that these results are based on genus‐level resolution, and the observed patterns may not directly reflect the dynamics of *Bacillus firmus* specifically, which constitutes the active component of the biological treatment. Genus‐level analyses can mask species‐ or strain‐specific responses, particularly in taxonomically diverse groups such as *Bacillus*, where introduced strains may represent only a small fraction of the total community. Importantly, the lack of sustained enrichment does not imply product inefficacy, but rather reflects the well‐documented ecological constraints on long‐term establishment of introduced strains under field conditions. Biological seed treatments may exert their effects through early‐stage interactions, functional activity, or indirect modulation of the rhizosphere rather than persistent increases in relative abundance. Together, these results indicate that *Bacillus* populations in field soils are resilient and primarily governed by temporal and site‐dependent factors, with biological inputs contributing to subtle, context‐dependent shifts consistent with realistic agroecosystem dynamics.

### 4.4 Stability and Compositional Shifts in Soil Bacterial and Fungal Communities

Microbial biodiversity in soil ecosystems is commonly assessed using alpha (α) and beta (β) diversity metrics, which capture complementary aspects of community structure. Alpha diversity reflects within‐sample richness and evenness and is typically quantified using the Shannon index (Cassol et al. 2025; Finn 2024; Walters and Martiny 2020; Pothula et al. 2022). Beta diversity describes differences in community composition among samples and was evaluated here using Bray–Curtis dissimilarity, which emphasizes shifts in relative abundance, and Jaccard dissimilarity, which captures qualitative turnover based on presence–absence data (Astudillo‐García et al. 2019; Machado et al. 2022; Walters and Martiny 2020).

Across locations and sampling times, alpha diversity analyses revealed high structural stability in both bacterial and fungal communities. No statistically significant differences in Shannon diversity were detected between control and treated samples, indicating that the combined treatment did not disrupt overall microbial richness or evenness under field conditions. Nevertheless, effect size estimates and directional trends suggest subtle, context‐dependent responses not captured by significance testing alone.

For bacterial communities, alpha diversity tended to be slightly higher in treated samples at the later sampling (S4), as indicated by moderate effect sizes, suggesting weak and temporally mediated responses. Similar patterns have been reported in systems where rhizosphere‐modifying interventions alter microbial interactions without inducing marked changes in overall diversity metrics (Vasantha Srinivasan et al. 2025; Walters and Martiny 2020; Zhao et al. 2023). These trends are consistent with gradual community reorganisation driven by plant development and soil–microbe feedbacks rather than direct selective pressure from the treatment.

Fungal alpha diversity showed a comparable but more consistent directional pattern, with higher mean Shannon values in treated samples at both sampling times, despite the absence of statistical significance. This contrasts with reports of fungicide‐induced fungal diversity loss and suggests community reorganisation rather than suppression, potentially reflecting differential tolerance among taxa and buffering by field‐level environmental heterogeneity (Delgado‐Baquerizo et al. 2016; Engelbrecht et al. 2018; Ristaino and Thomas 1997; Sartori et al. 2023).

Beta diversity responses were more pronounced than alpha diversity, highlighting the importance of compositional turnover. For bacterial communities, sampling time was the dominant driver of community differentiation across all locations, with ordination and PERMANOVA consistently showing stronger separation between early (S1) and late (S4) samples than between treatments. This temporal signal aligns with previous findings emphasising the role of plant phenology and seasonal dynamics in structuring soil bacterial communities (Lupwayi et al. 2019; Pothula et al. 2022; Walters and Martiny 2020).

Treatment effects on bacterial beta diversity were detectable but secondary and strongly location‐dependent, explaining a smaller proportion of variance than sampling time in most sites. These effects were primarily expressed as shifts in relative abundance, with limited qualitative turnover, consistent with the resilience and functional plasticity of bacterial assemblages under moderate disturbance (Streletskii et al. 2022).

In contrast, fungal beta diversity exhibited pronounced intrinsic heterogeneity, with pairwise Bray–Curtis and Jaccard dissimilarities revealing substantial differences in community composition across both space and time. However, PERMANOVA analyses indicated that neither treatment nor sampling time had statistically significant effects at any location, suggesting that observed variation primarily reflects natural fluctuations and local environmental heterogeneity rather than directional responses to the experimental interventions (Sahu et al. 2018; Timper 2014).

Overall, the absence of consistent alpha diversity losses combined with context‐dependent beta diversity shifts indicates that the treatment did not cause broad microbial disruption. Instead, it subtly modulated bacterial and fungal community trajectories within a framework largely governed by temporal succession and site‐specific conditions, supporting the view that soil microbiota under field conditions are resilient and capable of reorganisation following moderate chemical and biological interventions.

### 4.5 Study Limitations

This study presents some limitations that should be considered when interpreting the results. First, the field‐based experimental design, while enhancing ecological realism, involved a limited number of locations and composite samples, which constrained statistical power for several variables and required the use of non‐parametric and exploratory analyses. Second, *B. firmus* was applied exclusively in combination with a fungicide–insecticide seed treatment, preventing the complete disentanglement of biological and chemical effects, particularly regarding shifts in fungal communities and early plant responses. Third, microbial community analyses were based on relative abundance data derived from amplicon sequencing, which do not allow inference of absolute microbial biomass or population densities. Finally, the study was conducted over a single growing season, limiting conclusions about the long‐term persistence of treatment effects on soil microbiota, nematode populations, and crop performance. Future studies incorporating higher replication, factorial treatment designs, absolute quantification approaches, and multi‐season monitoring will be essential to refine the understanding of *B. firmus* performance under diverse agroecosystem conditions.

## 5 Conclusion

This study demonstrates that the application of a *Bacillus firmus* –based bionematicide in soybean production systems of the Pampa biome delivers consistent agronomic benefits under field conditions characterised by high ecological complexity. Across all evaluated environments, the treatment resulted in directional increases in grain yield (6%–7%), accompanied by pronounced and statistically robust enhancements in vegetative growth, particularly in shoot and root biomass. These results indicate a positive physiological effect on plant development, even in the absence of uniform nematode suppression.

Importantly, the observed agronomic gains were not associated with detectable changes in soil physicochemical properties or enzymatic activity, nor with consistent reductions in populations of target phytoparasitic nematodes. This pattern suggests that, under the conditions assessed, the primary contribution of the treatment was not direct nematode suppression or broad modification of soil fertility, but rather indirect promotion of plant performance. Such effects are likely mediated by transient or functional interactions within the rhizosphere that enhance plant efficiency and tolerance to biotic stress.

Metagenomic analyses further support this interpretation by showing that the seed treatment did not induce sustained enrichment of the genus *Bacillus* nor uniform restructuring of dominant bacterial communities. In contrast, fungal communities exhibited greater sensitivity to management practices, most likely reflecting the influence of the fungicide component of the seed treatment. Together, these findings indicate that the agronomic effectiveness of *B. firmus* ‐based products does not depend on persistent dominance within the soil microbiome, but instead emerges from subtle, context‐dependent interactions shaped by environmental conditions, plant phenology, and local soil characteristics.

The marked spatial variability observed among municipalities, with stronger responses under higher yield potential and greater water availability, underscores the critical role of environmental context in modulating the expression of biological inputs. These results challenge simplified expectations of uniform and direct nematode control in open‐field conditions and support a more integrative ecological perspective, in which bionematicides function as components of broader management strategies that enhance crop resilience and physiological performance rather than achieve pathogen eradication.

Overall, this work advances the understanding of bionematicide performance in realistic agricultural systems by demonstrating that *Bacillus firmus* can promote meaningful agronomic gains without causing major or persistent disruptions to soil microbial community structure or fully suppressing phytoparasitic nematodes. The results highlight the importance of integrating plant growth metrics, yield responses, and microbiome dynamics when evaluating biological inputs, and caution against relying solely on taxonomic abundance or pathogen suppression as indicators of efficacy. Future research should focus on elucidating the functional mechanisms underlying these responses—particularly plant physiological pathways, short‐term microbial interactions, and cumulative effects across growing seasons—to optimise the deployment of bionematicides within sustainable and site‐adapted management frameworks for soybean production in the Pampa biome.

## Author Contributions

**David Fagundes:** data curation, formal analysis, investigation, methodology, project administration, software, writing – original draft, writing – review and editing. **Laís Mara Santana Costa:** formal analysis, investigation, visualisation, writing – review and editing. **Alexandro Cagliari:** conceptualization, funding acquisition, investigation, methodology, project administration, supervision, visualisation. **Diego Prado de Vargas:** visualisation, writing – original draft, writing – review and editing. **Alexandre Rieger:** conceptualization, formal analysis, investigation, methodology, project administration, writing – original draft, supervision, writing – review and editing.

## Conflicts of Interest

The authors declare no conflicts of interest.

## Supporting information


**Appendix S1:** Supporting Information.

**Table S1:** Descriptive statistics of soybean yield (bags ha^−1^) for control (*n* = 10) and treated (*n* = 9) groups, reporting mean, standard deviation, minimum, and maximum values.
**Table S2:** Descriptive and statistical analysis of soil enzymatic activities and physicochemical properties for control and treated groups. The table presents group descriptives, including sample size (N), mean, standard deviation (SD), standard error (SE), and coefficient of variation for each variable. Comparisons between control and treated soils were performed using the Mann–Whitney U test, with U statistics, *p* values, and significance levels reported. Enzymatic analyses include arylsulfatase, β‐glucosidase, NAGase, and phosphatase activities, while physicochemical parameters include soil texture fractions, pH, macro‐ and micronutrients, cation exchange capacity (CEC), base and aluminium saturation, organic matter, and related indices. ns indicates no statistically significant difference between groups (*p* ≥ 0.05).
**Table S3:** Descriptive statistics of plant growth parameters for control and treated soybean plants across locations (Dom Pedrito, Candelária, and Camaquã) and sampling periods (Sampling 1 and 2). The table reports sample size (*n*), mean, standard deviation, and coefficient of variation for plant stand, shoot length, root length, shoot biomass, and root biomass in control and treated groups. Cells with missing values (NaN) indicate parameters not evaluated in a given location or sampling period.
**Table S4:** Relative abundance (%) of bacterial genera detected in soil samples from Dom Pedrito, Candelária, and Camaquã under control and treated conditions at two sampling periods (Sampling 1 and Sampling 4). Values represent the proportion of each genus within the total bacterial community per sample. Genera labelled as Unknown correspond to sequences not taxonomically assigned at the genus level. Totals sum to 100% for each sample.
**Table S5:** Statistical analysis of the relative abundance of dominant bacterial genera in soil samples across sampling periods (Sampling 1 and Sampling 4). For each genus, the table reports descriptive statistics, including sample size (*n*), mean relative abundance, standard deviation, and coefficient of variation for control and treated groups. Differences between paired control and treated samples were evaluated using the Wilcoxon signed‐rank test, with corresponding *p* values, significance levels, and effect sizes expressed as rank biserial correlation. ns indicates no statistically significant difference between groups (*p* ≥ 0.05).
**Table S6:** Relative abundance (%) of fungal genera detected in soil samples from Dom Pedrito, Candelária, and Camaquã under control and treated conditions at two sampling periods (Sampling 1 and Sampling 4). Values represent the proportion of each genus within the total fungal community per sample. Genera labelled as Unknown correspond to sequences not taxonomically assigned at the genus level. Totals sum to 100% for each sample.
**Table S7:** Statistical analysis of alpha diversity based on the Shannon index for bacterial and fungal communities across sampling periods (Sampling 1 and Sampling 4). The table presents descriptive statistics, including sample size (*n*), mean Shannon index, standard deviation, and coefficient of variation for control and treated groups. Paired comparisons between control and treated samples were performed using the Wilcoxon signed‐rank test, with corresponding *p* values, significance levels, and effect sizes expressed as rank‐biserial correlation. ns indicates no statistically significant difference between groups (*p* ≥ 0.05).
**Table S8:** PERMANOVA results for beta diversity of bacterial and fungal communities across locations (Dom Pedrito, Candelária, and Camaquã). The table reports the effects of treatment and sampling time on community composition, including Pseudo‐F statistics, proportion of explained variance (R^2^), *p* values, and significance levels. Residuals represent unexplained variance. Significance levels are indicated as *p* ≤ 0.05 (*), *p* < 0.01 (**), and ns for non‐significant effects.

## Data Availability

The datasets generated in this study are publicly available in online repositories. Raw sequence reads corresponding to the bacterial and fungal communities have been deposited in the NCBI database under BioProject accession number PRJNA1272157. Additional supporting information can be found online in https://doi.org/10.5281/zenodo.18175198.
