# Supplementary material for: Microbial Community Shifts and Plant Performance Improvements Driven by Bacillus firmus in Pampa Agroecosystems
Source: Environ Microbiol Rep. 2026 May 15;18(3):e70315. doi: 10.1111/1758-2229.70315 (PMC13179124; doi:10.1111/1758-2229.70315)
Supplement: Supplementary file 2 — Table S1: Descriptive statistics of soybean yield (bags ha−1) for control (n = 10) and treated (n = 9) groups, reporting mean, standard deviation, minimum, and maximum values. Table S2: Descriptive and statistical analysis of soil enzymatic activities and physicochemical properties for control and treated groups. The table presents group descriptives, including sample size (N), mean, standard deviation (SD), standard error (SE), and coefficient of variation for each variable. Comparisons between control and treated soils were performed using the Mann–Whitney U test, with U statistics, p values, and significance levels reported. Enzymatic analyses include arylsulfatase, β‐glucosidase, NAGase, and phosphatase activities, while physicochemical parameters include soil texture fractions, pH, macro‐ and micronutrients, cation exchange capacity (CEC), base and aluminium saturation, organic matter, and related indices. ns indicates no statistically significant difference between groups (p ≥ 0.05). Table S3: Descriptive statistics of plant growth parameters for control and treated soybean plants across locations (Dom Pedrito, Candelária, and Camaquã) and sampling periods (Sampling 1 and 2). The table reports sample size (n), mean, standard deviation, and coefficient of variation for plant stand, shoot length, root length, shoot biomass, and root biomass in control and treated groups. Cells with missing values (NaN) indicate parameters not evaluated in a given location or sampling period. Table S4: Relative abundance (%) of bacterial genera detected in soil samples from Dom Pedrito, Candelária, and Camaquã under control and treated conditions at two sampling periods (Sampling 1 and Sampling 4). Values represent the proportion of each genus within the total bacterial community per sample. Genera labelled as Unknown correspond to sequences not taxonomically assigned at the genus level. Totals sum to 100% for each sample. Table S5: Statistical analysis of the relative abundance [file EMI4-18-e70315-s002.pdf]

Supplementary Table_1

Supplementary Table 1 | Descriptive statistics of soybean yield (bags ha^{-1}) for control (n = 10) and treated (n = 9) groups, reporting mean, standard deviation, minimum, and maximum values.

YIELD (bags/hectare)		
	**Control**	**Treated**
**n**	10	9
**Mean**	59.05	63.67
**Std. Deviation**	16.78	18.46
**Minimum**	39.60	51.40
**Maximum**	91.10	100.10

Supplementary Table 2 | Descriptive and statistical analysis of soil enzymatic activities and physicochemical properties for control and treated groups. The table presents group descriptives, including sample size (N), mean, standard deviation (SD), standard error (SE), and coefficient of variation for each variable. Comparisons between control and treated soils were performed using the Mann–Whitney U test, with U statistics, p-values, and significance levels reported. Enzymatic analyses include arylsulfatase, β-glucosidase, NAGase, and phosphatase activities, while physicochemical parameters include soil texture fractions, pH, macro- and micronutrients, cation exchange capacity (CEC), base and aluminum saturation, organic matter, and related indices. *ns* indicates no statistically significant difference between groups ($p \geq 0.05$).

		Group Descriptives						*Mann-Whitney*		
		Treatment	N	Mean	SD	SE	Coefficient of variation	U	p	Significance (p < 0.05)
Enzymatic Analysis	Arylsulfatase	Control	12	262.7	170.82	49.31	0.650	80.00	0.671	ns
		Treated	12	231.2	153.13	44.20	0.662			
	Betaglucosidase	Control	12	262.7	136.69	39.46	0.520	63.00	0.630	ns
		Treated	12	339.9	230.76	66.61	0.679			
	NAGase	Control	12	129.3	63.47	18.32	0.491	63.00	0.630	ns
		Treated	12	146.5	85.39	24.65	0.583			
	Phosphatase	Control	12	1390.3	940.40	271.47	0.676	81.00	0.630	ns
		Treated	12	1172.0	700.82	202.31	0.598			
Physicochemical Analysis	Clay	Control	12	28.667	5.549	1.602	0.194	79.00	0.704	ns
		Treated	12	28.333	6.372	1.840	0.225			
	Sand	Control	12	45.250	16.249	4.691	0.359	52.50	0.268	ns
		Treated	12	47.750	15.755	4.548	0.330			
	Silt	Control	12	26.083	13.208	3.813	0.506	81.00	0.623	ns
		Treated	12	23.917	11.341	3.274	0.474			
	Texture	Control	12	3.000	0.426	0.123	0.142	61.00	0.350	ns
		Treated	12	3.167	0.389	0.112	0.123			
	pH	Control	12	5.525	0.214	0.062	0.039	89.50	0.319	ns
		Treated	12	5.383	0.369	0.106	0.069			
	P_mgL	Control	12	15.742	9.156	2.643	0.582	84.50	0.488	ns
		Treated	12	12.533	4.855	1.401	0.387			
	K_mgL	Control	12	160.167	79.448	22.935	0.496	70.00	0.932	ns
		Treated	12	157.167	66.787	19.280	0.425			
	K_perc	Control	12	2.937	1.193	0.345	0.406	64.50	0.686	ns
		Treated	12	3.153	1.098	0.317	0.348			
	SOM	Control	12	3.508	1.203	0.347	0.343	82.50	0.563	ns
		Treated	12	3.283	0.930	0.269	0.283			
	Ca_cmolcL	Control	12	8.109	3.788	1.094	0.467	89.50	0.326	ns
		Treated	12	6.730	2.990	0.863	0.444			
	Ca_per	Control	12	53.134	5.921	1.709	0.111	90.00	0.312	ns
		Treated	12	49.498	9.358	2.701	0.189			
	Mg_cmolcL	Control	12	2.900	1.017	0.293	0.351	78.50	0.729	ns
		Treated	12	3.410	2.913	0.841	0.854			
	CEC	Control	12	14.892	5.791	1.672	0.389	90.50	0.298	ns
		Treated	12	13.558	4.823	1.392	0.356			
	Base_saturation	Control	12	76.267	4.569	1.319	0.060	84.00	0.506	ns
		Treated	12	72.700	10.713	3.092	0.147			
	Al_Saturation	Control	12	0.417	0.609	0.176	1.461	60.50	0.496	ns
		Treated	12	1.308	3.085	0.891	2.358			
	S	Control	12	12.608	5.222	1.508	0.414	78.50	0.729	ns
		Treated	12	11.433	3.623	1.046	0.317			
	SMP	Control	12	6.283	0.393	0.113	0.063	75.50	0.862	ns
		Treated	12	6.233	0.485	0.140	0.078			
	Al_cmolcL	Control	12	0.050	0.067	0.019	1.348	60.50	0.479	ns
		Treated	12	0.117	0.221	0.064	1.893			
	Zn_mgL	Control	12	3.150	0.989	0.285	0.314	62.50	0.602	ns
		Treated	12	3.192	0.761	0.220	0.238			
	Cu_mgL	Control	12	1.500	0.453	0.131	0.302	90.50	0.296	ns
		Treated	12	1.308	0.458	0.132	0.350			
	B_mgL	Control	12	0.490	0.153	0.044	0.312	81.00	0.622	ns
		Treated	12	0.456	0.082	0.024	0.180			
	Mn_mgL	Control	12	22.000	8.801	2.541	0.400	52.00	0.405	ns
		Treated	11	25.909	12.988	3.916	0.501			
	Mg_per	Control	12	20.244	3.771	1.088	0.186	84.00	0.507	ns
		Treated	12	19.583	3.702	1.069	0.189			

Supplementary Table_3

Supplementary Table 3 | Descriptive statistics of plant growth parameters for control and treated soybean plants across locations (Dom Pedrito, Candelária, and Camaquã) and sampling periods (Sampling 1 and 2). The table reports sample size (n), mean, standard deviation, and coefficient of variation for plant stand, shoot length, root length, shoot biomass, and root biomass in control and treated groups. Cells with missing values (NaN) indicate parameters not evaluated in a given location or sampling period.

Location	Sampling	Descriptive statistics	Plant Stand		Shoot Length (cm)		Root Length (cm)		Shoot Biomass (g)		Root Biomass (g)	
			Control	Treated	Control	Treated	Control	Treated	Control	Treated	Control	Treated
Dom Pedrito	1	n	10	10	10	10	10	10	0	0	0	0
		Mean	10.9	11.1	12.8	13.3	10.3	10.9	NaN	NaN	NaN	NaN
		Std. Deviation	1.37	1.912	1.317	1.160	1.636	2.331				
		Coefficient of variation	0.126	0.172	0.103	0.087	0.159	0.214				
	2	n	10	10	10	10	10	10	10	10	10	10
		Mean	10.9	11.1	25.1	34.6	14.5	16.5	28.3	42.1	7.85	10.6
		Std. Deviation	1.37	1.913	1.853	3.893	2.068	2.068	1.054	4.006	0.158	1.476
		Coefficient of variation	0.126	0.172	0.074	0.113	0.143	0.125	0.037	0.095	0.02	0.139
Candelária	1	n	10	10	10	10	10	10	10	10	10	10
		Mean	10.8	12	15.9	20.4	11.8	14	9.95	13.650	2.4	3.1
		Std. Deviation	1.619	0.816	3.381	2.011	1.229	1.563	0.58	2.055	0.422	0.316
		Coefficient of variation	0.15	0.068	0.213	0.099	0.104	0.112	0.058	0.151	0.176	0.102
	2	n	10	10	10	10	10	10	10	10	10	10
		Mean	10.8	12	34	40.5	19.9	24.6	66.3	90.95	13.05	17.7
		Std. Deviation	1.619	0.816	3.496	1.179	5.087	3.596	2.214	0.896	0.896	0.527
		Coefficient of variation	0.15	0.068	0.103	0.029	0.256	0.146	0.033	0.01	0.069	0.03
Camaquã	1	n	10	10	10	10	10	10	10	10	10	10
		Mean	10.1	11.7	22.1	26.5	11.5	13.5	25.3	41.25	4.8	8.8
		Std. Deviation	1.663	1.418	2.644	1.9	2.068	1.780	0.527	5.745	0.211	1.687
		Coefficient of variation	0.165	0.121	0.12	0.072	0.18	0.132	0.021	0.139	0.044	0.192
	2	n	10	10	10	10	10	10	10	10	10	10
		Mean	10.1	11.7	33.8	43.1	16.7	23.2	75.35	137.9	21.350	30.45
		Std. Deviation	1.663	1.418	3.155	2.644	2.584	3.393	10.804	22.347	3.215	0.474
		Coefficient of variation	0.165	0.121	0.093	0.061	0.155	0.146	0.143	0.162	0.151	0.016

Supplementary Table_4

Supplementary Table 4 | Relative abundance (%) of bacterial genera detected in soil samples from Dom Pedrito, Candelária, and Camaquã under control and treated conditions at two sampling periods (Sampling 1 and Sampling 4). Values represent the proportion of each genus within the total bacterial community per sample. Genera labeled as *Unknown* correspond to sequences not taxonomically assigned at the genus level. Totals sum to 100% for each sample.

BACTERIAL GENERA	Dom Pedrito				Candelária				Camaquã			
	Sampling 1		Sampling 4		Sampling 1		Sampling 4		Sampling 1		Sampling 4	
	Control	Treated	Control	Treated	Control	Treated	Control	Treated	Control	Treated	Control	Treated
Unknown	31.6	43.4	41.2	36.5	43.4	47.0	43.0	42.9	41.7	41.1	44.2	39.6
Pseudomonas	18.7	0.0	0.0	0.0	0.0	0.0	0.0	0.0	0.0	0.0	0.0	0.0
Bacillus	13.9	14.1	25.4	22.7	17.8	11.8	16.6	17.7	3.8	6.7	5.4	5.0
Gaiella	10.0	8.9	6.3	6.6	2.7	3.1	4.2	5.3	4.2	5.2	3.9	6.8
Chthoniobacter	9.5	9.1	2.3	8.3	3.2	4.4	4.7	2.7	0.0	8.4	5.5	10.8
Conexibacter	3.1	2.9	2.0	2.0	2.7	2.6	3.4	3.0	3.0	2.3	3.0	3.4
Niallia	3.0	2.1	2.4	3.0	2.4	1.9	2.0	3.0	1.6	2.7	2.3	2.0
Neobacillus	2.8	3.3	3.3	4.2	5.3	4.9	4.6	6.5	1.9	3.9	2.8	2.9
Nitrososphaera	2.2	2.4	4.5	1.9	0.0	0.0	0.0	0.0	0.0	0.0	0.0	0.0
Bradyrhizobium	1.8	2.8	3.1	2.6	2.8	3.3	4.7	4.5	2.3	2.9	4.9	3.9
Spirillospora	1.7	2.1	1.3	1.4	1.9	2.3	2.1	2.2	0.0	0.0	0.0	0.0
Koribacter	1.6	3.4	2.6	2.8	4.0	5.8	3.7	4.0	4.5	2.9	4.9	3.5
Nitrosocosmicus	0.0	1.4	1.7	1.5	1.8	2.1	2.0	1.8	0.0	0.0	0.0	0.0
Pedosphaera	0.0	1.4	0.0	0.0	0.0	1.8	0.0	0.0	0.0	1.8	0.0	1.7
Luteitalea	0.0	2.7	2.5	1.7	3.9	1.8	2.9	0.0	1.9	2.5	3.6	3.1
Ureibacillus	0.0	0.0	1.4	1.8	2.3	0.0	1.7	2.1	0.0	0.0	0.0	0.0
Peribacillus	0.0	0.0	0.0	1.3	0.0	0.0	0.0	0.0	0.0	0.0	0.0	0.0
Sphingomicrobium	0.0	0.0	0.0	1.7	1.6	1.6	2.5	2.4	2.4	2.3	2.9	3.3
Tumebacillus	0.0	0.0	0.0	0.0	1.4	1.4	0.0	0.0	0.0	0.0	0.0	0.0
Pyrinomonas	0.0	0.0	0.0	0.0	1.4	0.0	0.0	0.0	0.0	0.0	0.0	0.0
Flavisolibacter	0.0	0.0	0.0	0.0	1.4	0.0	0.0	0.0	0.0	0.0	0.0	0.0
Acidobacterium	0.0	0.0	0.0	0.0	0.0	1.5	0.0	0.0	2.3	1.9	0.0	1.9
Thermosporothrix	0.0	0.0	0.0	0.0	0.0	1.4	0.0	0.0	2.1	0.0	0.0	0.0
Streptomyces	0.0	0.0	0.0	0.0	0.0	1.4	1.9	0.0	0.0	0.0	0.0	0.0
Sporosarcina	0.0	0.0	0.0	0.0	0.0	0.0	0.0	1.5	0.0	0.0	0.0	0.0
Methanocella	0.0	0.0	0.0	0.0	0.0	0.0	0.0	0.0	19.0	10.6	7.7	4.8
Methanothrix	0.0	0.0	0.0	0.0	0.0	0.0	0.0	0.0	2.3	0.0	0.0	0.0
Methanosarcina	0.0	0.0	0.0	0.0	0.0	0.0	0.0	0.0	1.9	0.0	0.0	0.0
Mycobacterium	0.0	0.0	0.0	0.0	0.0	0.0	0.0	0.0	1.9	1.5	1.8	2.1
Pseudarthrobacter	0.0	0.0	0.0	0.0	0.0	0.0	0.0	0.0	1.6	0.0	1.6	1.5
Methyloceanibacter	0.0	0.0	0.0	0.0	0.0	0.0	0.0	0.0	1.5	1.6	2.1	1.9
Priestia	0.0	0.0	0.0	0.0	0.0	0.0	0.0	0.0	0.0	1.9	0.0	0.0
Rhodomicrobium	0.0	0.0	0.0	0.0	0.0	0.0	0.0	0.0	0.0	0.0	1.9	0.0
Roseiarcus	0.0	0.0	0.0	0.0	0.0	0.0	0.0	0.0	0.0	0.0	1.7	0.0
Nocardioides	0.0	0.0	0.0	0.0	0.0	0.0	0.0	0.0	0.0	0.0	0.0	1.7
**TOTAL**	100.0	100.0	100.0	100.0	100.0	100.0	100.0	100.0	100.0	100.0	100.0	100.0

Supplementary Table_5

Supplementary Table 5 | Statistical analysis of the relative abundance of dominant bacterial genera in soil samples across sampling periods (Sampling 1 and Sampling 4). For each genus, the table reports descriptive statistics, including sample size (n), mean relative abundance, standard deviation, and coefficient of variation for control and treated groups. Differences between paired control and treated samples were evaluated using the Wilcoxon signed-rank test, with corresponding p-values, significance levels, and effect sizes expressed as rank-biserial correlation. *ns* indicates no statistically significant difference between groups ($p \geq 0.05$).

Group	Sampling	Descriptive statistics	Relative abundance		Wilcoxon signed-rank test		
			Control	Treated	p value	significance	Rank-bisserial correlation
Bacillus	1	n	3	3	1	ns	0
		Mean	5.541	5.447			
		Std. Deviation	1.281	0.712			
		Coefficient of variation	0.231	0.131			
	4	n	3	3	0.5	ns	0.667
		Mean	5.885	5.696			
		Std. Deviation	1.149	1.202			
		Coefficient of variation	0.195	0.211			
Gaiella	1	n	3	3	0.75	ns	0.333
		Mean	4.837	4.755			
		Std. Deviation	1.262	0.945			
		Coefficient of variation	0.261	0.199			
	4	n	3	3	0.75	ns	-0.333
		Mean	4.849	4.991			
		Std. Deviation	0.599	0.326			
		Coefficient of variation	0.123	0.065			
Chthoniobacter	1	n	3	3	0.75	ns	-0.333
		Mean	2.088	5.045			
		Std. Deviation	5.484	0.768			
		Coefficient of variation	2.626	0.152			
	4	n	3	3	0.5	ns	-0.667
		Mean	4.659	4.995			
		Std. Deviation	0.228	0.377			
		Coefficient of variation	0.049	0.076			
Conexibacter	1	n	3	3	0.25	ns	1
		Mean	4.332	4.060			
		Std. Deviation	0.686	0.512			
		Coefficient of variation	0.158	0.126			
	4	n	3	3	0.5	ns	0.667
		Mean	4.313	4.167			
		Std. Deviation	0.277	0.447			
		Coefficient of variation	0.064	0.107			
Bradyrhizobium	1	n	3	3	0.75	ns	-0.333
		Mean	4.080	4.203			
		Std. Deviation	0.489	0.370			
		Coefficient of variation	0.12	0.088			
	4	n	3	3	0.5	ns	0.667
		Mean	4.719	4.442			
		Std. Deviation	0.203	0.564			
		Coefficient of variation	0.043	0.127			

Supplementary Table_6

Supplementary Table 6 | Relative abundance (%) of fungal genera detected in soil samples from Dom Pedrito, Candelária, and Camaquã under control and treated conditions at two sampling periods (Sampling 1 and Sampling 4). Values represent the proportion of each genus within the total fungal community per sample. Genera labeled as *Unknown* correspond to sequences not taxonomically assigned at the genus level. Totals sum to 100% for each sample.

FUNGAL GENERA	Dom Pedrito				Candelária				Camaquã			
	Sampling 1		Sampling 4		Sampling 1		Sampling 4		Sampling 1		Sampling 4	
	Control	Treated	Control	Treated	Control	Treated	Control	Treated	Control	Treated	Control	Treated
Fusarium	21.6	15.8	10.6	9.6	19.3	13.5	13.1	5.5	9.4	4.0	8.2	6.9
Unknown	20.6	10.8	24.9	11.5	27.8	28.4	24.1	20.9	17.1	20.0	20.4	11.4
Fusicolla	15.0	39.2	4.2	0.0	0.0	0.0	0.0	0.0	8.1	0.0	0.0	0.0
Mortierella	11.4	1.3	4.0	5.1	2.4	2.8	12.0	12.0	16.7	30.9	23.3	7.9
Gongronella	8.9	0.0	0.0	0.0	0.0	1.7	0.0	1.3	0.0	0.0	0.0	0.0
Clonostachys	6.6	2.7	4.2	29.7	2.4	1.4	2.6	1.9	0.0	1.6	3.5	21.9
Neocosmospora	5.1	0.0	0.0	0.0	0.0	1.7	0.0	5.2	0.0	0.0	0.0	0.0
Penicillium	4.1	5.8	2.6	5.9	11.4	3.3	5.4	4.7	9.3	8.0	8.8	7.4
Humicola	3.0	0.0	2.7	2.3	0.0	0.0	0.0	0.0	0.0	0.0	0.0	0.0
Gibberella	2.1	15.2	0.0	0.0	2.9	4.3	6.4	0.0	0.0	0.0	0.0	0.0
Rhizopus	1.6	4.4	0.0	0.0	0.0	4.2	2.4	0.0	0.0	0.0	0.0	0.0
Plectosphaerella	0.0	3.7	1.5	0.0	1.6	2.2	2.5	2.8	0.0	2.7	0.0	3.8
Ciliophora	0.0	1.2	0.0	0.0	0.0	0.0	0.0	0.0	2.7	0.0	2.6	1.3
Alternaria	0.0	0.0	16.9	12.7	1.3	1.4	0.0	0.0	0.0	0.0	0.0	0.0
Xepicula	0.0	0.0	16.6	9.6	0.0	0.0	0.0	0.0	0.0	0.0	4.3	0.0
Cladosporium	0.0	0.0	7.3	8.4	0.0	0.0	1.8	1.5	0.0	0.0	1.2	3.2
Fusariella	0.0	0.0	2.5	2.3	0.0	0.0	0.0	0.0	0.0	0.0	0.0	0.0
Talaromyces	0.0	0.0	2.0	1.2	3.0	15.5	6.0	12.5	0.0	0.0	0.0	0.0
Gibellulopsis	0.0	0.0	0.0	1.7	8.4	5.7	11.3	7.1	0.0	0.0	0.0	0.0
Aspergillus	0.0	0.0	0.0	0.0	7.4	3.5	0.0	0.0	4.7	4.9	3.8	2.7
Purpureocillium	0.0	0.0	0.0	0.0	3.2	1.3	1.7	0.0	0.0	0.0	0.0	0.0
Trichoderma	0.0	0.0	0.0	0.0	2.2	4.2	2.2	1.8	0.0	2.8	1.2	0.0
Chloridium	0.0	0.0	0.0	0.0	1.6	2.2	1.2	2.4	0.0	0.0	0.0	0.0
Calvatia	0.0	0.0	0.0	0.0	1.4	0.0	0.0	0.0	0.0	0.0	0.0	0.0
Beauveria	0.0	0.0	0.0	0.0	1.3	0.0	0.0	0.0	0.0	0.0	0.0	0.0
Westerdykella	0.0	0.0	0.0	0.0	1.2	1.3	2.0	0.0	4.5	1.9	1.7	1.6
Zopfiella	0.0	0.0	0.0	0.0	1.2	0.0	0.0	0.0	0.0	0.0	0.0	0.0
Podospora	0.0	0.0	0.0	0.0	0.0	1.2	0.0	0.0	0.0	0.0	0.0	0.0
Sesquicillium	0.0	0.0	0.0	0.0	0.0	0.0	2.6	0.0	0.0	0.0	0.0	0.0
Cadophora	0.0	0.0	0.0	0.0	0.0	0.0	1.5	1.2	0.0	0.0	0.0	0.0
Atractiella	0.0	0.0	0.0	0.0	0.0	0.0	1.2	0.0	0.0	0.0	0.0	0.0
Knufia	0.0	0.0	0.0	0.0	0.0	0.0	0.0	1.2	0.0	0.0	0.0	0.0
Vishniacozyma	0.0	0.0	0.0	0.0	0.0	0.0	0.0	17.8	0.0	0.0	0.0	0.0
Talaromyces	0.0	0.0	0.0	0.0	0.0	0.0	0.0	0.0	8.0	4.5	5.2	3.7
Pseudeurotium	0.0	0.0	0.0	0.0	0.0	0.0	0.0	0.0	5.3	1.7	1.2	1.6
Saitozyma	0.0	0.0	0.0	0.0	0.0	0.0	0.0	0.0	3.3	2.1	3.3	3.1
Lycoperdon	0.0	0.0	0.0	0.0	0.0	0.0	0.0	0.0	3.3	1.1	0.0	0.0
Schizothecium	0.0	0.0	0.0	0.0	0.0	0.0	0.0	0.0	2.9	5.9	3.1	0.0
Coniochaeta	0.0	0.0	0.0	0.0	0.0	0.0	0.0	0.0	1.6	3.5	2.8	1.3
Massariosphaeria	0.0	0.0	0.0	0.0	0.0	0.0	0.0	0.0	1.6	1.7	1.9	0.0
Rhizophlyctis	0.0	0.0	0.0	0.0	0.0	0.0	0.0	0.0	1.5	0.0	0.0	0.0
Pyrenochaetopsis	0.0	0.0	0.0	0.0	0.0	0.0	0.0	0.0	0.0	2.7	2.0	0.0
Candida	0.0	0.0	0.0	0.0	0.0	0.0	0.0	0.0	0.0	0.0	1.7	0.0
Periconia	0.0	0.0	0.0	0.0	0.0	0.0	0.0	0.0	0.0	0.0	0.0	2.6
Albifimbria	0.0	0.0	0.0	0.0	0.0	0.0	0.0	0.0	0.0	0.0	0.0	19.5
**TOTAL**	100.0	100.0	100.0	100.0	100.0	100.0	100.0	100.0	100.0	100.0	100.0	100.0

Supplementary Table_7

Supplementary Table 7. Statistical analysis of alpha diversity based on the Shannon index for bacterial and fungal communities across sampling periods (Sampling 1 and Sampling 4). The table presents descriptive statistics, including sample size (n), mean Shannon index, standard deviation, and coefficient of variation for control and treated groups. Paired comparisons between control and treated samples were performed using the Wilcoxon signed-rank test, with corresponding p-values, significance levels, and effect sizes expressed as rank-biserial correlation. *ns* indicates no statistically significant difference between groups (p ≥ 0.05).

Group	Sampling	Descriptive statistics	Shannon index		Wilcoxon signed-rank test		
			Control	Treated	p value	significance	Rank-bisserial correlation
Bacteria	1	n	3	3	0.75	ns	0.333
		Mean	2.060	2.027			
		Std. Deviation	0.050	0.150			
		Coefficient of variation	0.024	0.074			
	4	n	3	3	0.586	ns	-0.500
		Mean	2.077	2.110			
		Std. Deviation	0.11	0.154			
		Coefficient of variation	0.053	0.073			
Fungi	1	n	3	3	0.5	ns	-0.667
		Mean	2.313	2.380			
		Std. Deviation	0.195	0.156			
		Coefficient of variation	0.084	0.066			
	4	n	3	3	0.5	ns	-0.667
		Mean	2.173	2.297			
		Std. Deviation	0.312	0.127			
		Coefficient of variation	0.143	0.055			

Supplementary Table_8

Supplementary Table 8. PERMANOVA results for beta diversity of bacterial and fungal communities across locations (Dom Pedrito, Candelária, and Camaquã). The table reports the effects of treatment and sampling time on community composition, including Pseudo-F statistics, proportion of explained variance (R^2), p-values, and significance levels. Residuals represent unexplained variance. Significance levels are indicated as $p \leq 0.05$ (*), $p < 0.01$ (**), and *ns* for non-significant effects.

Group	Location	Factor	Pseudo-F	R²	p-value	significance
Bacteria	Dom Pedrito	Treatment	1.92	0.13	0.040	*
		Sampling time	3.45	0.24	0.010	**
		Residuals	–	0.63	–	
	Candelária	Treatment	1.68	0.14	0.050	*
		Sampling time	2.74	0.19	0.030	*
		Residuals	–	0.67	–	
	Camaquã	Treatment	2.51	0.21	0.020	*
		Sampling time	1.98	0.16	0.040	*
		Residuals	–	0.63	–	
Fungi	Dom Pedrito	Treatment	0.17	0.08	1.000	ns
		Sampling time	2.64	0.57	0.339	ns
		Residuals	–	0.35	–	
	Camaquã	Treatment	1.51	0.43	0.354	ns
		Sampling time	1.19	0.37	0.689	ns
		Residuals	–	0.20	–	
	Candelária	Treatment	0.38	0.16	1.000	ns
		Sampling time	1.38	0.41	0.651	ns
		Residuals	–	0.43	–	
